# *Sparus aurata* and *Lates calcarifer* skin microbiota under healthy and diseased conditions in UV and non-UV treated water

**DOI:** 10.1186/s42523-022-00191-y

**Published:** 2022-06-21

**Authors:** Ashraf Al-Ashhab, Rivka Alexander-Shani, Yosef Avrahami, Roberto Ehrlich, Rosa Ines Strem, Shiri Meshner, Noam Shental, Galit Sharon

**Affiliations:** 1 Dead Sea and Arava Science Center, 8698000 Masada, Israel; 2 Ben Gurion University of the Negev, Eilat Campus, Beersheba, Israel; 3 Israel Oceanographic and Limnological Research Ltd., The National Center for Mariculture, Eilat, Israel; 4 Computer Science Department, The Open University of Israel, Rehovot, Israel

**Keywords:** Fish skin, Fish microbiome, Bacterial fish diseases, *Sparus aurata*, *Lates calcarifer*

## Abstract

**Background:**

The welfare of farmed fish is influenced by numerous environmental and management factors. Fish skin is an important site for immunity and a major route by which infections are acquired. The objective of this study was to characterize bacterial composition variability on skin of healthy, diseased, and recovered Gilthead Seabream (*Sparus aurata*) and Barramundi (*Lates calcarifer*). *S. aurata*, which are highly sensitive to gram-negative bacteria, were challenged with *Vibrio harveyi*. In addition, and to provide a wider range of infections, both fish species (*S. aurata* and *L. calcarifer*) were infected with gram-positive *Streptococcus iniae*, to compare the response of the highly sensitive *L. calcarifer* to that of the more resistant *S. aurata*. All experiments also compared microbial communities found on skin of fish reared in UV (a general practice used in aquaculture) and non-UV treated water tanks.

**Results:**

Skin swab samples were taken from different areas of the fish (lateral lines, abdomen and gills) prior to controlled infection, and 24, 48 and 72 h, 5 days, one week and one-month post-infection. Fish skin microbial communities were determined using Illumina iSeq100 16S rDNA for bacterial sequencing. The results showed that naturally present bacterial composition is similar on all sampled fish skin sites prior to infection, but the controlled infections (T_1_ 24 h post infection) altered the bacterial communities found on fish skin. Moreover, when the naturally occurring skin microbiota did not quickly recover, fish mortality was common following T_1_ (24 h post infection). We further confirmed the differences in bacterial communities found on skin and in the water of fish reared in non-UV and UV treated water under healthy and diseased conditions.

**Conclusions:**

Our experimental findings shed light on the fish skin microbiota in relation to fish survival (in diseased and healthy conditions). The results can be harnessed to provide management tools for commercial fish farmers; predicting and preventing fish diseases can increase fish health, welfare, and enhance commercial fish yields.

**Supplementary Information:**

The online version contains supplementary material available at 10.1186/s42523-022-00191-y.

## Background

Population growth, increased fish consumption, the great contribution of fish to food security, and social development have made aquaculture the fastest growing food sector globally with an average of 8% annual increase over the last 30 years [1, 2]. Yet, this annual increase is expected to double in the coming decade [2]. The increased production rate is accompanied by many environmental problems including disease outbreaks of many fish-related pathogens [3]. Intensive fish rearing practices, increased production and continuous stress conditions in aquariums compromise fish health, immunity, and increase susceptibility to infection [4]. Stress can be broadly defined as a state in which a series of adaptive responses reestablish homeostasis following exposure to a stressor [5, 6]. In fish, stress responses include activation of the hypothalamus–pituitary–internal (HPI) axis, culminating in the release of glucocorticoids from internal cells in the head kidney [7]. In intensive aquaculture, farmed fish are frequently exposed to stressors such as crowding and handling, which influence health and welfare, and can threaten aquaculture sustainability [8, 9]. In natural settings, fish populations are increasingly becoming subjected to multiple anthropogenic stressors, which threaten their sustainability [10–12]. Stress-mediated impairment of immune function has been widely described in cultured and wild fish, and is associated with an increased susceptibility to disease [13–15]. Mucosal immune response plays a crucial role in the course of the infection and includes healthy and dynamic microbial communities [16–19]. In fact, some bacteria are fish species-specific [20] and early studies have shown that some fish skin bacteria have beneficial roles in excreting friction-reducing polymers [21], in governing fish behavior and communication [22], and in fighting other pathogenic bacteria [23]. The evidence of bacterial importance in fish skin has stressed that both skin bacteria and skin function should be investigated as one entity referred to as a holobiont. Recently, research groups have investigated the relations between fish skin microbiome and skin ulcer infections in aquaculture of Atlantic salmon [24], and in parasitic copepod *Lepeophtheirus salmonis* [25]. Others reviewed the importance of fish skin microbiota under stress conditions and during antibiotic application [26–28]. Studies describing the concept of maintaining healthy fish microbiota [29] have stressed that future studies should scrutinize the specific mechanisms by which different members of the fish microbiota and the metabolites they produce interact with pathogens, with other commensals, and with immunity responses. This objective has driven researchers to develop fish gut probiotics that control growth performance, specific growth rate, weight gain, final weight, feed conversion ratio, immunity and bacterial infection [30–33].

In this study, we characterized the variability of skin bacterial composition in healthy and diseased gilthead seabream (*Sparus aurata*) and barramundi (*Lates calcarifer*). Using a high-throughput DNA sequencing method we compared fish skin (abdomen, lateral lines, and gills) microbiome in a controlled environment, in UV and non-UV treated water, in healthy and diseased fish infected with an opportunistic pathogen gram negative bacterium (*Vibrio harveyi*) and a true pathogen, gram positive bacteria (*Streptococcus iniae*) both can infect many fish species.

## Methods

### Experimental setup

All the experiments were conducted in a quarantine facility at the NCM institute, located adjacent to the Red Sea in Eilat Israel. Sea water from the Red Sea is pumped and directed to an AGF 48" sand filtration of > 20 µm filtration (Arkal, Israel) entering the NCM institute, after which water goes through UV treatment at the entrance of the quarantine facility (see Additional file 2 for Sea water physical, chemical and biological characteristics during experimentation).

*Sparus aurata*: Twenty 150 ± 10 g fish were stocked in two separate 100 l tanks and placed in a stabilized environment (10 fish per tank) under continuous flow of either UV or non-UV treated seawater (40 ppt). This experiment was repeated twice, in the end of summer (September 2015) and in winter (January 2016), assessing 40 fish overall. Fish were infected with *Vibrio harveyi* bacteria in both experiments. Under the same conditions, an additional experiment was conducted on twenty 60 ± 5 g *S. aurata* fish infected with *Streptococcus iniae* in January 2020 (Table 1).

**Table 1:** Experimental design showing different treatments and sampling points

Exp	Date	Tank/treatment	Experimental fish	Introduced pathogen
1	13–16, Sep 2015	UV water	S*. aurata* (swabs were not processed)	*V. harveyi*
	13–27, Sep 2015	Non-UV water	S*. aurata* (abdomen, gills, and lateral lines)	
2	11–14, Jan 2016	UV water	S*. aurata* (abdomen, gills, and lateral lines)	
	11–27, Jan 2016	Non-UV water		
3	Jan 15th–Apr 12th 2020	UV water	S*. aurata* (lateral lines)	*S. iniae*
		Non-UV water		
4		UV water	*L. calcarifer* (lateral lines)	
		Non-UV water		

*Lates calcarifer*: Twenty 55 ± 5 g barramundi (*L. calcarifer*) fish were stocked in two separate 100 l tanks (10 fish per tank) under a continuous flow of either UV or non-UV treated seawater (40 ppt) and were infected with *S. iniae* (January 2020).

Before the onset of experiments, fish (*S. aurata* and *L. calcarifer*) from all treatment groups (UV and non-UV) were acclimated to the environment for two weeks in separate tanks. Throughout all different experiments fish were fed 2% of their body weight daily using Raanan Fish Feed Ltd. (Oshrat, Israel; 3 mm feed with 46/18% protein/fat ratio for Marine fish).

### Fish tagging and sampling

Fish were anesthetized using clove oil (25 µl/L for 10 min until loss of movement, followed by 12.5 µl/L for continued anesthesia supplemented with aeration). Each fish was assigned a different serial number by injecting a subcutaneous (S.C) P-tag (Trovan). Fish from each treatment group were sampled using a sterile cotton swab (FLOQSwabs in tube ® 553C-COPAN) during the tagging process at the beginning of the experiment (T_0_) and at each time point as described below.

For the *V. harveyi* bacterial infection experiments, samples were taken by swabbing a ~ 1 cm^2^ area of fish skin mucous layer. One swab was taken from each area: abdomen (A), gills (G; taken from the right filaments between the first and second gill arch), and right-side lateral line (L). Samples were collected at the beginning of the experiment (T_0_), 24 h after stress and exposure to pathogen (T_1_), after 1 week (T_2_) and after 3 or 5 weeks (T_3_-week 3 in the winter experiment and week 5 in the summer experiment) (Table 1).

For the *S. iniae* infection experiment, samples were taken only from the lateral line. Samples were taken during the tagging process at the beginning of the experiment (T_0_), and 24 h (T_1_), 48 h (T_1–2_), 72 h (T_1–3_), 5 days (T_1–5_), 1 week (T_2_), 2 weeks (T_2–2_) and 1 month (T_3_) post infection (Table 1).

After sampling, each swab was inserted into a clean, sterile, and dry test tube and was kept at – 80 °C until analysis. Fish were monitored daily for signs of disease. In addition, 500 ml samples of water from the fish tanks were filtered through a 0.22 µm filter paper (Macherey–Nagel (MN) USA) at the set time points, with an additional sampling point at 60 min after infection (Ts). All samples were kept at − 80 °C until used.

### Pathogenic bacterial culture and application

*Vibrio harveyi*, and *Streptococcus iniae* were obtained from the National Center for Mariculture (NCM) pathology department from the bacterial stocks kept at − 80 °C. *V. harveyi*, was originally isolated from spleen of *Sparus aurata* in 2012 and *S. iniae* was originally isolated from the liver of a *Siganus rivulatus* in 2010. Both bacteria were sent for 16S rRNA sequencing at Hy Laboratories Ltd., and were identified, compared, and aligned with those of other *V. harveyi*, and *S. iniae* available in the GenBank database (NCBI/BLAST) before further use. Bacteria from the -80 °C were then defrosted to room temperature and inoculated in a laminar flow hood on tryptic soy agar (TSA, DIFCO USA) prepared with 25% sterile seawater, and incubated at 24 ± 1 °C for 48–72 h. After the incubation period, the bacterial isolates were transferred to tryptic soy broth (TSB, ACUMEDIA USA) prepared with 25% sterile seawater and incubated again for another 48–72 h at 24 ± 1 °C. OD values from the bacterial concentration were read at 600 nm using a microplate spectrophotometer (PowerWave^TM^XS, BioTek, Winooski, USA).

### Stress implementation, infection, and fish monitoring

After fish tagging (described in Sect. 2.1), stress was implemented to magnify the impact of the bacterial infection as follows; Fish were netted out of the water for 5 min (handling stress), and then subjected to a needle scratch on their caudal fin by a sterile (23 G) needle. Immediately afterwards, fish were immersed in a *Vibrio harveyi* bacterial suspension (250,000 bacterium / ml) in a reduced water tank volume (5 l). After 60 min of immersion, the water tank was gradually refilled to its initial volume of 100 l within one hour. After a 24 h recovery period, first samples were taken (T_1_). All bacterial infections were done the same way except for a small modification for *S. iniae*. In the *S. iniae* trial, fish were transferred to an aerated container with 5 l of seawater containing bacterial suspension at a concentration of 5 × 10^7^ CFU bacteria/l for 10 min and returned to their respective tanks. Fish were monitored daily throughout the experiments for signs of disease; fish showing clinical signs were recorded and freshly dead fish were sampled for bacteriological analysis to resolve disease etiology. Mortality rates from the different treatment groups in all experiments were recorded.

### DNA extraction, library preparation and Illumina sequencing

Swab samples taken from different treatments at different time points were individually clipped under sterile conditions and set up for DNA extraction using the MoBio 96-well plate PowerSoil DNA Isolation Kits (MO BIO Laboratories, California, USA), following the manufacturer's protocol. All steps of DNA extraction were carried out in a sterile UV-hood (DNA/RNA UV-cleaner box, UVT-S-AR bioSan, Ornat, Israel) to reduce external contaminations. In every DNA extraction, 200 µl of RNase free water was used as a negative control (Sigma Aldrich, Israel). All samples were placed randomly in the DNA extraction plate to exclude any bias.

For the *V. harveyi* infection experiment (Table 1), in order to increase phylogenetic resolution and diversity estimates, a multiplex PCR using five different sets of the 16S rDNA genes was applied to cover about 1,000 bp of the 16S rRNA gene [34] (Additional file 3: Table S1). First PCR (PCR I) reactions were performed in triplicates, where each PCR-I reaction (total 25 µl) contained: a) 12.5 µl of KAPA HiFi HotStart ReadyMix (biosystems, Israel); b) 0.4 µl of equal v/v mixed primers forward and reverse primers; c) 10 µl of molecular graded DDW (Sigma, Israel); and d) 2 µl of (2–100 ng/µl) DNA template. PCR I reactions were performed in Biometra thermal cycler (Biometra, TGradient 48) as follows: initial denaturation at 95 °C for 2 min, followed by 35 cycles of 98 °C for 10 s, 61 °C for 15 s, and 72 °C for 7 s. The PCR I routine ended with a final extension at 72 °C for 1 min. Upon completion of PCR I, we ran an electrophoresis gel to verify that all samples were successfully amplified. Following successful and verified amplification, triplicate samples were pooled together and cleaned using Agencourt® AMPure XP (Beckman Coulter, Inc, Indianapolis, USA) bead solution following the manufacturer’s protocol.

Library preparation was performed using a second PCR (PCR II) to connect the Illumina linker, adapter and unique 8 base pair barcode for each sample [34]. The PCR II reactions were prepared by mixing 21 µl of KAPA HiFi HotStart ReadyMix (biosystems, Israel), 2 µl of mixed primers with Illumina adapter (Additional file 3: Table S2), 12.6 µl of RNase free water (Sigma, Israel), and 4 µl of each sample from the first PCR product with 2 µl of barcoded reverse primer. This was placed in Biometra thermal cycler (Biometra, TGradient 48) as follows: initial denaturation 98 °C for 2 min, and then 8 cycles of 98 °C for 10 s, 64 °C for 15 s, 72 °C for 25 s, and a final extension of 72 °C for 5 min. Then all PCR II products were pooled together and cleaned using Agencourt® AMPure XP (Beckman Coulter, Inc., Indianapolis, USA) bead solution following manufacturer’s protocol, where 50 µl of pooled PCR II product were cleaned using 1:1 ratio with the bead solution for more conservative size exclusion of fragments less than 200 bp, and at the final step, 50 µl of DDW with 10 mM Tris [pH = 8.5] were added to each sample. This was followed by aliquoting 48 µl of the supernatant to sterile PCR tubes and storing in -80 °C, while an additional 15 µl of the final product was sent to the Hebrew University (Jerusalem, Israel) and sequenced on full lane of 250 bp paired end reads (to correct for sequencing errors and enhance total read quality) using Illumina MiSeq platform.

For the *S. iniae* infection experiment (Table 1), we used V4-16S rDNA F515 and R806, [35] and its related Illumina primers (Additional file 3: Table S3) for PCR I and PCR II using the same aforementioned protocols and procedures, however, DNA samples were sequenced using 150 bp paired-end reads using Illumina iSeq100 platform at our laboratories.

### Sequence curation and quality control

First, the *V. harveyi* infection experiment sequences were filtered for PhiX using Bowtie2 [36], then incomplete, low-quality reads (phred Q threshold 33) and incomplete paired sequences were removed using PEAR software [37]. Following the previous quality control steps, sequences were analyzed using QIIME-2 software [38]. In QIIME-2, sequences were aligned, checked for chimeric sequences and clustered to different OTUs (operational taxonomic unit) based on 99% sequence similarity, then classified based on Greengenes database V13.8 [39]. The generated OTU table was also cleared from sequences classified as f_mitochondria, o_Chloroplast, k_Archaea and K_Unclassified, those removed sequences accounted for less than 1.2% of total obtained sequences number (including removal of OTU’s having less than 10 aligned sequences). Both the number of raw sequences and bacterial classified sequences were recorded in Additional file 3: Table S1 and the third primer set (F649-R889) was selected as representative for the microbial community composition (see Additional file 2, Data validation).

After the *S. iniae* infection experiment in 2020, the sequences (F649-R889) from the earlier *V. harveyi* infection experiment (summer 2015 and winter 2016) were curated and analyzed together as follows: first, samples were filtered for primer sequences, then sequence errors were cleared with MAX_CONSIST = 20 and repeated sequences were removed. Then sequences were clustered using DADA2 [40], and paired-end sequences were merged with minimum overlapping of 20 base pairs. After merging, samples were cleaned from chimeric sequences, the sequences were assigned to taxonomical classification using Silva database V138 with 99% sequence similarity [41] and an ASV table was generated. A similar analytic procedure was performed for the *S. iniae* infection experiment sequences; however, we first produced the paired-end sequences (to obtain similar fragment length as in the *V. harveyi* experiment) using PEAR, and then we followed the same protocol.

### Data curation and analysis

Data curation: Both generated ASV tables (2015 + 2016 and 2020 experiments) were curated as follows: only sequences classified in the kingdom Bacteria were maintained, then sequences classified as *NA_Phylum*, *Chloroplastes_Order* and *Mitocondria_Family* were removed from both ASV tables (accounting for 24.5% of total obtained sequences). Then only samples having a total sequence number of over 1000 sequences were maintained for downstream analysis. Following initial data curation, additional filters were applied to remove noise, for example, we removed low read ASVs (≤ 10 reads) (Additional file 3: Table S4). Afterwards, a rarefactions curve was produced (Additional file 1: Fig. S1).

Data analysis: Non phylogenetic alpha diversities, including (A) Chao1 species’ diversity estimate [42], (B) Shannon diversity [43], (C) Simpson diversity index [44] were calculated using the VEGAN package in R [45]. Faith’s phylogenetic diversity [46] was calculated from the curated dataset using the PhyloMeasures package in R [47]. After determining alpha diversity, we compared beta diversity among groups and treatments. To investigate the absolute and weighted “abundance” of shared ASVs, we generated different Venn diagrams using the “eulerr” package in R [48]. Then PCoA dissimilarity ordination plots were generated based on weighted unifrac distance matrix explaining beta diversity variations among the different treatments and temporal scales. Significance tests were performed for the various treatments using single or pairwise comparisons using permutational multivariate analysis of the variance (adonis) based on Bray–Curtis distance matrix [49] using pairwise.adonis function in R with Bonferroni correction for adjusting p values using 999 permutations. Taxonomic distribution graphs were generated based on the ASV tables; each phylum was assigned a distinct color and all genera under the same phylum were assigned different shades of the same color.

## Results

### Effect of UV and non-UV treated water on fish survival

**Fig. 1:**
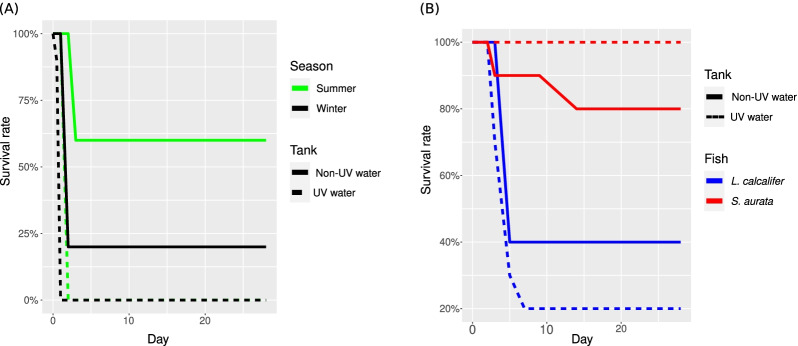
Survival rate in percentage over 30 days post infection of *S. aurata* after *V. harveyi* infection in summer 2015 and winter 2016 (**A**) and for Both *L. calcarifer* and *S. aurata* fish species after *S. iniae* infection in 2020, winter season (**B**)

For the V. harveyi infection experiments, fish mortality was recorded daily (Fig. 1). Dead fish were removed from the experiment and subjected to bacteriological analysis to confirm mortality etiology. Figure 1a shows that *S. aurata* fish reared in non-UV treated water had significantly higher survival rates following *V. harveyi* infection (60% survival in summer and 20% in winter), compared to fish reared in UV treated water (no survivors in either season).

In our second round of experiments, we assessed *S. iniae* bacterial infection in the less susceptible *S. aurata* and more susceptible *L. calcarifer* fish (Fig. 1b). For the *S. iniae* infection, the survival rate of *L. calcarifer* increased from 20% in UV treated water to 40% in non-UV treated water. The survival of *S. aurata* infected with *S. iniae* in UV treated water was 100% compared to 80% in non-UV treated water.

### Commensal bacterial diversity at different spatial and temporal changes following pathogenic bacterial infection

**Fig. 2:**
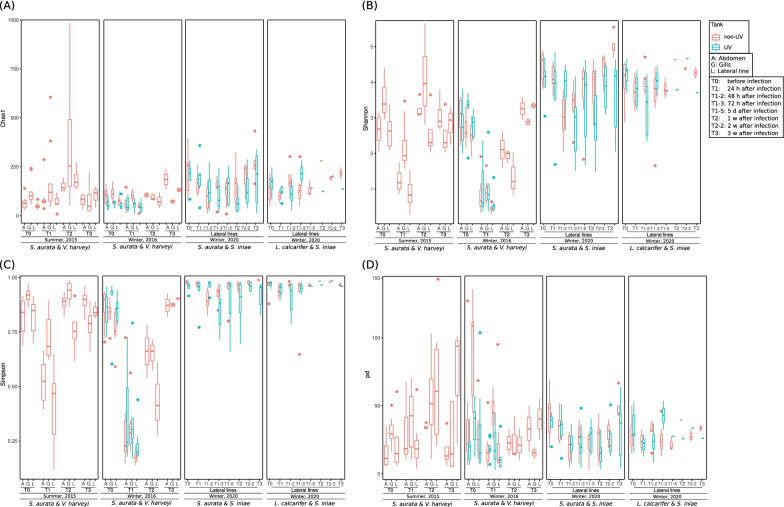
Chao1 (**A**), Shannon (**B**), Simpson’s (**C**) and Faith’s phylogenetic bacterial diversity (**D**) indices for *S. aurata* abdomen (A), gills (G) and lateral lines (only *L. calcarifer)* (L) in UV (light blue) and non-UV (red) tanks at different time points before infection (*V. harveyi* and *S. iniae*) (T_0_), and post-infection (T1–T_3_)

Fish skin bacterial diversity estimates, including non-phylogenetic Chao1 species’ diversity, Shannon and Simpson diversity index, and Faith’s phylogenetic diversity (Fig. 2) all showed a slightly higher diversity for non-UV treated water compared to UV treated water. Interestingly, during the infection (T_1_), we saw a remarkable decrease in fish skin microbial diversity estimates of both *V. harveyi* and *S. iniae* pathogens and these diversities returned to their initial level at T_2_ and T_3_, corresponding to one week and one month post infection. Figure 2 presents diversity estimates at the different body sites (abdomen, gills and lateral) during *V. harveyi* infection in the summer 2015 and winter 2016 experiments. At different time points, we noticed a higher similarity in those diversity estimates for both fish abdomen and lateral lines compared to gills which showed slightly higher estimates, however these differences showed to be insignificant when compared using Tukey’s test (Additional file 1: Fig. S2).

**Fig. 3:**
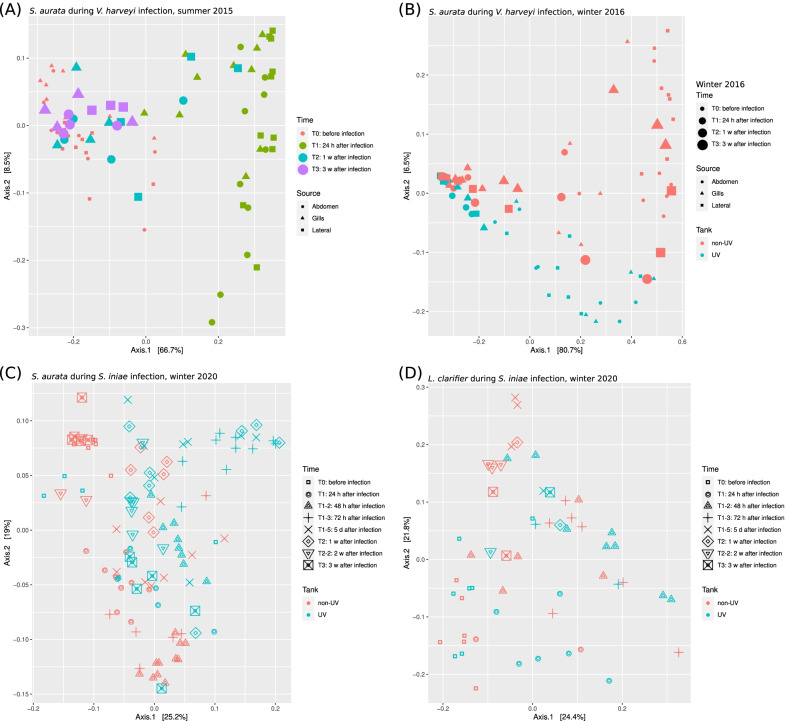
PCoA ordination plots based on weighted Unifrac distance matrix for different experiments. PCoA plots for *S. aurata* during *V. harveyi* infection, summer 2015 season (**A**) and winter 2016 season (**B**). **C** and **D** Shows PCoA plots during *S. iniae* infection for *S. aurata* (**C**) and *L. calcarifer* (**D**) in 2020 experiments

To better illustrate these differences and to evaluate related patterns in the bacterial communities, PCoA ordination plots based on weighted unifrac distance matrix were generated for the different experiments. Figure 3a–d shows PCoA plots for *S. aurata* during *V. harveyi* infection (Fig. 3a,b) in summer 2015 and in winter 2016 respectively, while Fig. 3c and d shows PCoA plots during *S. iniae* infection for *S. aurata* (Fig. 3c) and *L. calcarifer* (Fig. 3d).

Figure 3 and the pairwise statistical differences (Additional file 3: Table S5) show distinctly unique fish skin bacterial communities in fish from UV and non-UV tanks for *S. aurata* during both *V. harveyi* (Fig. 3b) and *S. iniae* infection (Fig. 3c) but not for *L. calcarifer* (Fig. 3d, Additional file 3: Table S5). Interestingly, *S. aurata* only showed a significant difference in community composition when comparing the water treatments at T_0_ but not at T_1_ for the *V. harveyi* infection. However, during the *S. iniae* infection, significant differences in bacterial communities were evident when comparing the UV and non-UV tanks at all time points. Note, there were no significant differences in the bacterial composition of gills at T_0_ when comparing UV and non-UV treatments for *S. aurata* during the *V. harveyi* infection (Additional file 3: Table S5).

When looking at the shared and unique microbial ASVs, only 21, 31 and 25% of all ASVs are shared between both UV and non-UV treatments for winter, 2016 V*. harveyi* infection and for 2020 *S. iniae* infection in *S. aurata* and *L. calcarifer* respectively, these also constitute 93, 93 and 85% of weighted bacterial abundances, respectively (Additional file 3: Table S6). In contrast, the percent and weighted percentages of the shared and unique microbial communities were similar among the different time points and body sites. Notice, fish skin microbial communities in non-UV treatment had a higher percentage of unique ASVs compared to fish reared in UV treated tanks; this percentage declined during infection (Additional file 3: Table S6B) and gradually increased post infection. Interestingly, the number of shared ASVs has shown to positively correlate with disease severity and negatively correlate with survival rate (Fig. 1).

When comparing the microbiota of different fish body sites (abdomen, gills, and lateral line), Fig. 3a and b do not show a clear separation. When testing the significant differences between fish body sites at different time points in UV and non-UV treatment (Additional file 3: Table S7), microbial communities do show significant differences but only at a few time points. Differences in microbial communities are evident when comparing the microbial communities of the lateral line to gills at T_0_ and T_1_ in the non-UV treatment in both summer and winter experiments, and once again when abdominal microbial communities were compared at T_1_ in the summer experiment (Additional file 3: Table S7A). The unweighted and weighted percentages of unique ASVs for different body sites at different time points for non-UV treatments (Additional file 3: Table S7B), clearly indicate the presence of different microbial communities when comparing gills and lateral line sites, reaching up to 50% unique ASVs at T_2_ for the gills site.

Figure 3a and b also show samples of microbial community variance at different time points. The most pronounced separation is indicated by axis 1 and explains 66.7% of microbial variance in relation to different time points. These microbial variances are mainly seen at T_1_ (24 h post infection), while axis 2 only explained 8.5% of the variation which corresponded to the changes accruing between UV and non-UV treatments during summer season in 2015 (Fig. 3a). When this experiment was repeated in winter 2016 (Fig. 3b), axis 1 explained 80.7% of total bacterial variation at the different time points before, during and after infection. In both winter and summer experiments, before (T_0_) and after infection (T_1_), all body sites for both UV and non-UV treatments showed to be significantly different (Additional file 3: Table S8A). Interestingly, the microbial community on skin of surviving fish returned to its original composition two weeks post infection (comparing between T_0_ and T_3_, *P*-values > 0.05). When infecting both *S. aurata* (Fig. 3c) and *L. calcarifer* fish species with *S. iniae* (Fig. 3d), we attempted to monitor changes in the microbial communities at higher temporal resolution to understand their interactions and impacts on fish health. Therefore, additional sampling time points were added at 48 h (T_1–2_), 72 h (T_1–3_), 5 days (T_1–5_), 1 week (T_2_), and 2 weeks (T_2–2_) post infection. Differences in the microbial communities between the UV and non-UV treatments were observed (Fig. 3c, Additional file 3: Table S5); in addition, the PCoA plot presented in Fig. 3c also shows interesting temporal patterns. The microbial communities showed a gradual deviation from T_0_ downward along axis 2 (explaining 19% of variance) for samples taken at T_1_ and T_1-2_ (24 h and 48 h after infection, respectively), while at T_1–3_ (72 h after infection) the microbial communities began to return to the original composition like T_1_ (Additional file 3: Table S8B). Interestingly, after five days (T_1–5_), one week (T_2_), two week (T_2–2_) microbial communities gradually moved to cluster with T_3_ (one month after infection) which was like the original microbial communities (*P*-value = 0.056 between T_0_ and T_3_). In the UV treatment, there were no significant differences at the different time points compared to T_0_ (Additional file 3: Table S8C), yet a significant difference was observed comparing different stages of infection (T_1–2_, T_1–3_, T_1–5_, T_2_, T_2–2_ and T_3_), quite like differences seen in the non-UV treatment.

During *S. iniae* infection of *L. calcarifer,* which is a highly susceptible fish species (see Fig. 1b), T_0_ did not show a clear separation of the microbial communities or statistical differences between the non-UV and UV treatments (Additional file 3: Table S5). There were no clear differences at different time points neither before nor after infection in both non-UV and UV treatments (Additional file 3: Table S8D and S8E), except when comparing bacterial communities after 24 (T_1_) and 72 h (T_1–3_) post infection. Interestingly, following *V. harveyi* (Fig. 3a,b) and *S. iniae* (Fig. 3c,d) infections, a major difference in the variance of the microbial communities was seen in the PCoA analysis, explaining 87.2 and 75.2% of variance in *V. harveyi* while for *S. iniae* infection, explained 44.2 and 46.2% of variance for axis 1 and 2 respectively.

### Bacterial community compositions

**Fig. 4:**
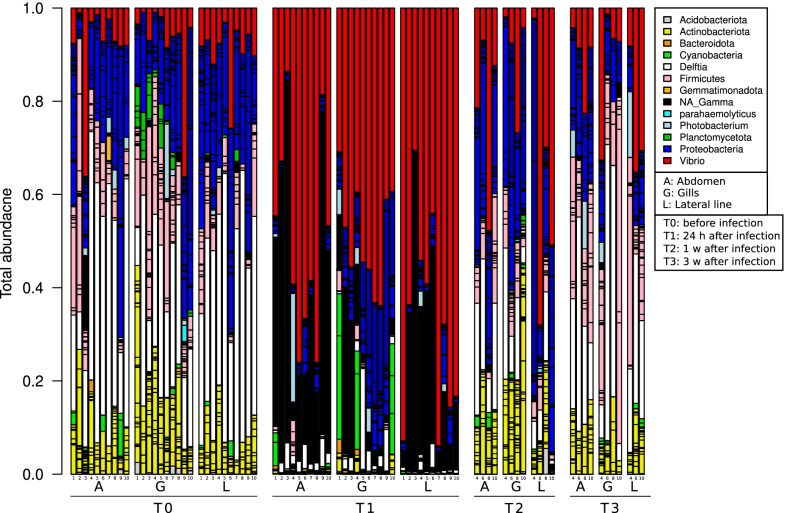
Bar graph illustrating relative abundances of different bacterial phyla (different colors). Each bar represents one (tagged) fish at different grown in the non-UV water tank at time points (T_0_ before infection, T_1_ 24 h, T_2_ one week, T_3_ three weeks post infection), body sites (Abdomen (A); Gills (G); Lateral line (L)) for non-UV treatment, during *V. harveyi* infection in summer, 2015

**Fig. 5:**
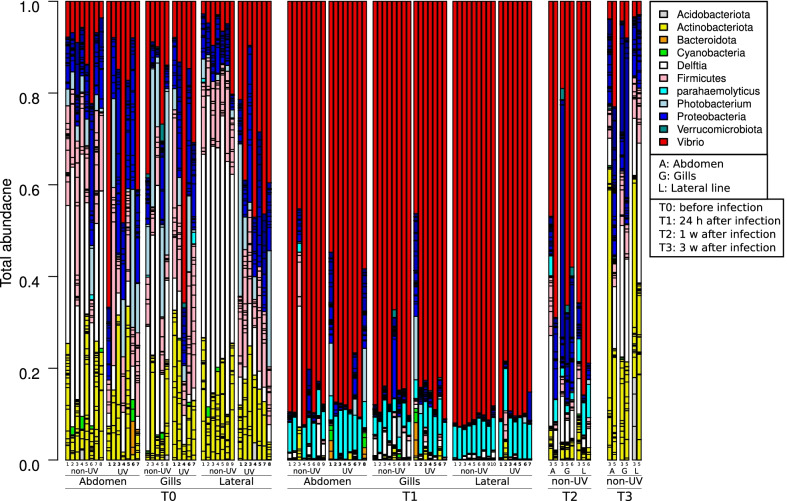
Bar graph illustrating relative abundances of different bacterial phyla (different colors). Each bar represents one (tagged) fish, at different time points (Before infection T_0_ and 24 h T_1_; one-week T_2_; three-weeks T_3_ post infection), body sites for both UV and non-UV treatments during *V. harveyi* infection in winter, 2016

**Fig. 6:**
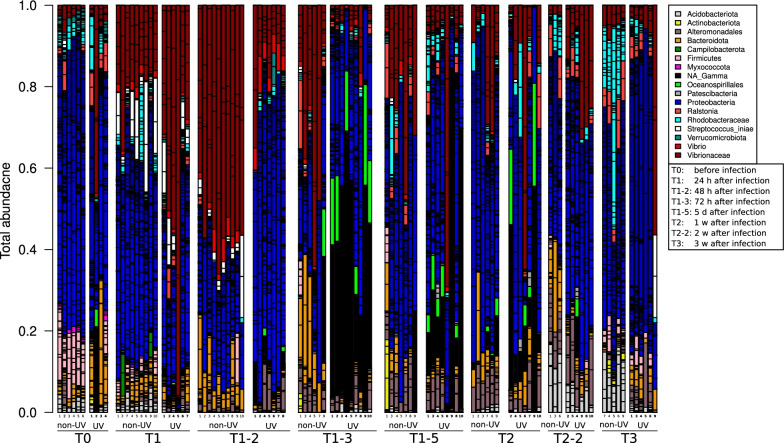
Bar graph illustrating relative abundances of different bacterial phyla (different colors). Each bar represents one (tagged) fish, at different time points (T_0_- before infection, T_1_ 24 h, T_1-2_ 48 h, T_1-3_ 72 h, T_1-5_ five days, T_2_ one week, T_2-2_ two weeks and T_3_ three weeks post infection), for UV and non-UV treatments during *S. iniae* infection in *S. aurata*

**Fig. 7:**
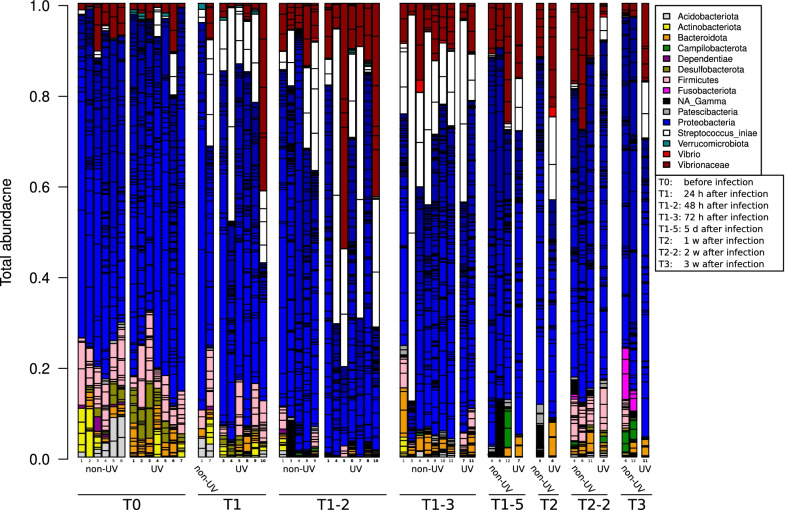
Bar graph illustrating relative abundances of different bacterial phyla (different colors). Each bar represents one (tagged) fish at different time points (T_0_ before infection, T_1_ 24 h, T_1–2_ 48 h, T_1–3_ 72 h, T_1–5_ five days, T_2_ one week, T_2–2_ two weeks and T_3_ three weeks post infection), for UV and non-UV treatments during *S. iniae* infection in *L. calcarifer*

Figures 4, 5, 6 and 7 show the relative abundance of each bacterial phylum (different shades of the same color present different families of the same phyla) during *V. harveyi* and *S. iniae* infection experiments. Figure 4 illustrates the relative bacterial abundance during *V. harveyi* infection in the summer 2015 experiment; the bar graph shows three main bacterial phyla dominating the total bacterial abundance, *Proteobacteria* (blue, red, and white), *Firmicutes* (pink) and *Actinobacteria* (yellow). At T_0_, before infection, *Proteobacteria* abundance was 63.9 ± 12.2%, followed by *Firmicutes* (14.8 ± 11.0%) and *Actinobacteria* (13.1 ± 8.1%). Following infection, these relative bacterial abundances changed at T_1_ (24 h after infection) and T_2_ (1 week after infection) and T_3_ (3 weeks after infection) yet they were still dominant and the final relative abundances at T_3_ were 56.6 ± 6.3% for *Proteobacteria*, 27.1 ± 19.0% for *Firmicutes* and 11.0 ± 4.9% for *Actinobacteria*. To better understand the changes in relative bacterial abundances and their effect on fish health before, during and after infection, we analyzed the relative bacterial abundances to pinpoint their significant changes at different sampling points using DeSeq (Additional file 1: Fig. S3). DeSeq analysis showed seven ASVs to significantly differentiate at different time points. The most abundant ASV belongs to the *Unclassified_Gammaproteobacteria Class* ASV of *Proteobacteria (Gray,* Additional file 1: Fig. S3) which mainly dominated T_1_ (24 h post infection) at a relative abundance of 24.1 ± 22.3%*,* followed by *Delftia* ASV genus at T_0_ (33.9 ± 15.3%). Interestingly, *Unclassified_Gammaproteobacteria* ASV was only abundant at T_1_, (24 h post infection). At one- and three-weeks post infection (T_2_ and T_3_), its relative abundance declined to less than 1% of the total bacterial abundance and was replaced with *Delftia* ASV; their relative abundances were 11.2 ± 8.2% and 24.2 ± 16.9% respectively (Fig. 4).

When repeating the same experiment in winter 2016, we analyzed relative bacterial abundances in both UV and non-UV treated tanks (Fig. 5). Figure 5 shows the similar three main bacterial phyla dominating the total bacterial abundance: *Proteobacteria*, *Firmicutes and Actinobacteria*. The relative abundances of these bacterial phyla proved to be different in the non-UV and UV treatment tanks. At T_0_, before infection, *Proteobacteria* abundance in non-UV versus UV treated water was 57.3 ± 6.5% and 53.5 ± 9.1% respectively, followed by *Actinobacteria* (18.2 ± 9.6% and 15.6 ± 9.8%) and *Firmicutes* (15.1 ± 7.1% and 20.1 ± 9.3%). DeSeq analysis shows that the abundance of ten bacterial ASVs significantly differentiate in different UV treatments and time points *Delftia* and *Bacillus* genus of *Proteobacteria* and *Firmicutes* are among the most abundant ASVs (Additional file 1: Fig. S4)*.* Interestingly, *Delftia* (white) and *Bacillus* (yellow) showed a significantly different distribution following UV treatment at T_0_: the abundance of both *Delftia* and *Bacillus* decreased from 33.9 ± 15.3% to 4.9 ± 2.4%, and from 5.6 ± 3.6 to 0.2 ± 0.5% respectively. At T_1_, 24 h after infection with *V. harveyi*, the bacterial community was dominated by the *Vibrio* family (red), with a relative abundance of 85.0 ± 10.4% and 80.6 ± 13.7% for non-UV and UV treated water, respectively (Fig. 4). Moreover, *Delftia* genus also showed a higher abundance in non-UV treated (2.9 ± 5.2%) compared to UV treated water (0.3 ± 0.3%), while *Photobacterium (*cyan*,* belonging to *Vibrio* family*)* showed a higher abundance in UV treated (1.9 ± 3.9%) compared to non-UV treated water (0.2 ± 0.8%) (Additional file 1: Fig. S4). At T_2_ (one-week post-infection) and T_3_ (three weeks post-infection), all fish from UV treated water perished; in non-UV treated tanks, the relative abundance of *vibrio* decreased to 58.9 ± 20.7% at T_2_ and to 6.6 ± 6.5% at T_3_ (Fig. 4). On the other hand, *Delftia* increased from 6.9 ± 5.0% to 16.6 ± 8.7% at T_2_ and T_3_ respectively and the final relative abundances of the main bacterial phyla were 50.4 ± 10.9%, 35.1 ± 14.8% and 9.7 ± 2.9% for *Proteobacteria*, *Actinobacteria* and *Firmicutes* respectively, similar to the initial abundance at T_0_. Interestingly, the *Delftia* ASV was dominant at T_0_ for both summer (33.9 ± 15.3%, Fig. 4), and winter experiment (29.5 ± 15.1%; Fig. 5), whereas at T_1_, *Delftia* relative abundances significantly declined to 2.1 ± 3.9% and bacterial communities were dominated by *Vibrio* ASV abundance (53.5 ± 20.7%). However, a remarkable increase in the *Unclassified_Gammaproteobacteria* abundance at T_1_ was only observed in the summer experiments (24.1 ± 22.3%, Additional file 1: Fig. S3).

The results of the pathogenic *S. iniae* infection experiment conducted in 2020 (Fig. 6) show that bacterial communities of *S. aurata* fish skin at T_0_ (before infection) are slightly different compared to those at T_0_ in the previous experiments (summer 2015 and winter 2016; Figs. 4 and 5). The most abundant bacterial phyla in this experiment were *Proteobacteria*, *Firmicutes* and *Bacteroidota* with relative T_0_ abundances of 67.3 ± 4.3% and 66.1 ± 13.7%, 11.7 ± 3.2% respectively for the non-UV treatment, and 2.4 ± 3.2%, and 4.2 ± 0.8% and 14.8 ± 12.3% respectively for the UV treated water. *Actinobacteria,* previously seen in the two *V. harveyi* experiments as one of the three main bacterial phyla, was less abundant (2.3%) during this experiment (Fig. 6). Interestingly, at T_1_ (24 h post-infection) of the *S. iniae* infection (white*,* Fig. 6), the relative abundance of *S. iniae* did not dominate fish skin lateral line (unlike fish infected with *V. harveyi*) and showed a relative abundance of 9.0 ± 8.6% and 5.9 ± 4.5% for non-UV and UV treatments, respectively. Yet, DeSeq analysis showed *Unclassified_Vibrionaceae* family ASV (brown, Additional file 1: Fig. S5), and *Unclassified_Gammaproteobacteria Class* ASV (black, Additional file 1: Fig. S5), to significantly differentiate at different time points and between UV and non-UV treatment. *Unclassified_Vibrionaceae* Family showed an increased abundance from 2.4 ± 1.6% at T_0_ to 18.3 ± 6.3% at T_1_ for non-UV treatment and from 13.7 ± 20.9% at T_0_ to 44.5 ± 27.6% at T_1_ for UV treated water. At T_1-2_ (72 h after infection), *Unclassified_Vibrionaceae* Family abundance further increased to reach 51.4 ± 7.4% for non-UV treated water. In addition, following the infection, *Unclassified_Gammaproteobacteria* (black) ASV showed an increased abundance at the recovery stage (T_1-2_, T_1-3_, T_1-5_ and T_2_), however its abundance was higher in the UV treatment compared to non-UV treated water (Additional file 1: Fig. S5).

The *S. iniae* infection experiment was also conducted on *L. calcarifer* fish (Fig. 7). At T_0_, before infection with *S. iniae*, the main bacteria phyla found on the lateral line of *L. calcarifer* were *Proteobacteria*, *Firmicutes* and *Bacteroidota* at relative abundances of 70.3 ± 3.6% and 72.8 ± 6.5% for non-UV treatment respectively, and 10.9 ± 2.2% and 8.2 ± 4%, 5.1 ± 2.3% and 7.3 ± 2.9% for UV treated water, respectively. At T_1_ (24 h post infection), there was a similar yet higher increase in *S. iniae* abundance in the skin (lateral line) of *L. calcarifer* compared to *S. aurata*. The relative abundances for *S. iniae* at T_1_, T_1-2_ and T_1-3_ for non-UV treated water were 10.0 ± 11.1%, 10.8 ± 9.8% and 27.5 ± 25.6% while for UV treated water, they were 16.5 ± 12.4%, 27.6 ± 22.1% and 23.3 ± 19.7% respectively (Fig. 7). DeSeq analysis showed the abundance of *Unclassified_Rhodobacteraceae Family* ASV to be significantly higher on fish skin before infection (T_0_), 24 h (T_1_) and 72 h (T_1-2_) post infection for non-UV treated water compared to UV treated water (Additional file 1: Fig. S6).

### Microbiota differences between individual tagged fish

To investigate the differences in fish skin microbiota between the different individual tagged fish, we used pairwise.adonis function in R for multivariate analysis of the variance Bray–Curtis distance matrix. No significant differences (*P*-values > 0.05) were noticed in skin microbiota between the different tagged fish within the groups, as well as to those fish that did not survive infection. Moreover, there were no differences found at different time points for both UV and non-UV tanks nor for different sampled body sites in both fish species (Additional file 3: Table S9).

## Discussion

Aquaculture is one of the fastest growing food sectors [1, 2]. Recent studies highlighted the emergence of different fish pathogens within this sector's fast growing industry [3, 4, 9, 50–55]. Stress-mediated impairment of immune functions has been widely described in cultured and wild fish causes increased susceptibility to disease [13–15, 52, 53, 56–59]. While some papers have investigated fish skin microbiome [19, 20, 23, 53, 60, 61], published studies on fish microbial dysbiosis and disease development are limited [24, 27, 29, 62, 63]. The results presented in this paper describe innate occurring fish skin microbiota during healthy, diseased and recovery conditions in the presented experemintal setup. As well as comparison between different fish body sites (abdomen, gills, and lateral lines), different seasons (summer and winter), and fish reared in UV treated water (a normal practice in fish farms) and non-UV treated water. The experimental setups also explored pathogenic infections of *V. harveyi* (gram negative) and *S. iniae* (gram positive) bacteria in two different fish species, *S. aurata* and *L. calcarifer* (two fish species grown for food in the aquaculture industry of the Middle East). Comparisons were verified using a set of quality controls and different technical replicates based on the V4 region of the 16S rRNA using illumina iSeq 100 platform (Additional file 1: Fig. S1). Notice following quality control steps some of the samples failed to reach the sequencing plateau in the number of obtained ASV which is to consider either by lowering the amount of samples per iSeq sequeing lane or changing the sequencing platform to obtain more throughput reads per sample.

### Microbial diversity, composition, and survival rate of fish in UV and non-UV treated water post infections

We compared fish skin microbiota in a controlled environment, in UV and non-UV treated water, in healthy and diseased fish. In most experiments, fish reared in UV treated water showed significantly reduced survival following bacterial infection (Fig. 1). As expected, infections by *S. iniae,* a gram-positive bacterial pathogen, caused up to 20% mortality in the less susceptible *S. aurata*, compared to up to 80% mortality and higher disease severity in the highly sensitive *L. calcarifer* (Fig. 1a,b). Similarly, comparably high mortality rates were documented previously with *Vibrio* spp. infection in *S. aurata* and *Dicentrarchus labrax* fish species [64, 65]. Many researchers have suggested that water disinfection, whether by UV, ozonation or ultrasonication, is an essential practice in aquaculture that prevents pathogenic infections in fish [66–69]. Here we compared similar conceptual comparisons and the most closely related paper was recently published in 2021 by Attramadal et al. [70, 71] who investigated the effect of UV treatment on lobster larvae survival, showing results similar to ours. Their study showed 43% enhanced larva survival in tanks subjected to recirculating aquaculture system (RAS) without UV compared to those reared in RAS with UV treatment, without introducing any stress or pathogenic infection to lobster larvae [70]. Their results strengthen our findings of higher diversity indices (both Shannon and Species richness “Chao1”) when comparing non-UV and UV treated water (Fig. 2).

The higher fish survival rate in the non-UV treatment following infection may be attributed to the stability of microbial communities [72]. A recent paper [73] investigated lumpfish (*Cyclopterus lumpus* L.) in UV vs non-UV treated water. Using histopathological analysis, it showed the improved gill health of fish reared in non-UV treated water. Dahle et al. [73] suggested that disinfecting water (using UV) may reduce overall fish growth, gill health, and increase fish mortality. Our study showed that skin shared microbiota form between 21 and 31% of total microbial communities (Additional file 3: Table S6). These percentages accounted for: 93, 93 and 85% of the total community abundances found in the second (*S. aurata* infected with *V. harveyi* in winter 2016), third (*S. aurata* infected with *S. iniae* winter 2020) and fourth (*L. calcarifer* infected with *S. iniae*) experiments respectively (Additional file 3: Table S5). In fact, when comparing between UV and non-UV-treatments, we noticed a significant difference in microbial communities at T_0_ (before infection) for both *S. aurata* and *L. calcarifer*. When *S. aurata* was infected with *V. harveyi* and when *L. calcarifer* was infected with *S. iniae*, no significant differences were observed in fish from either UV treatment following infection. However, significant differences between the two UV treatments were seen when *S. aurata* was infected with *S. iniae* at all the sampling points (Additional file 3: Table S5, Fig. 3). Both *V. harveyi* and *S. iniae* bacteria are pathogens for wild and cultured fish and can cause a wide range of symptoms and even death [74]. Yet, *S. iniae* pathogen is less virulent to *S. aurata* compared to *L. calcarifer*. Our results show that *S. iniae* dominated the *L. calcarifer* skin microbiota after infection with no significant difference after the bacterial infection, however, with *S. aurata*, *S. iniae* was less dominant on fish skin after bacterial infection. These differences remained throughout the experiment on fish reared in UV-treated water tanks.

According to the ecological theory of r/K-selection (MacArthur and Wilson, 1967), selective pressures (i.e. stress, induction or UV treatment) drive microbial succession either by selection for opportunists (r-selection), or for specialists (K-selection). When fish are subjected to UV treatment, the high resource supply per bacterium favors the fast-growing species (r-selection). Therefore, different bacterial communities, which were present at T_0_, significanly changed when a strong pathogen that was introduced managed to dominate fish skin microbiota. However, when the fish reacted to a less virulent pathogen (in the case of *S. aurata* infected with *S. iniae*) K-selection strategies were maintained, thus lowering mortality rates. This notion was discussed by Vestrum et al. [71], who showed that different water treatment systems induced differences in larval microbiota. This observation indicates that non-stress conditions promote K-selection and microbial stability by maintaining a microbial load close to the carrying capacity of the system [71].

### Spatial variation in fish skin microbiome from different body sites

No significant variations were noticed in the microbiota of samples taken from different body sites of *S. aurata*, (Fig. 3). However, at T_0_ and T_1_, diversity estimates showed that bacterial community of the gills is slightly higher in Shannon, Simpson, and Faith’s phylogenetic diversity, compared to fish skin abdomen and lateral line areas (Fig. 2). Investigating pairwise significance, only the microbial communities of the gills significantly differentiated at specific time points, namely when compared with lateral lines at T_0_ and T_1_ (Additional file 3: Table S7A). Looking at the unique ASVs (Additional file 1: Figs. S3 and S4) at T_0_ and T_1_, up to 53% of the total microbial ASVs were unique for the gills despite being only 9% of the weighted abundance at most (Additional file 3: Table S7B). Moreover, gills are a thin barrier between fish blood and the environment [75]. This sophisticated system has a large surface area and delicate structure that provide an ideal port of entry for molecules, particles, and all kinds of pathogens [76]. As such, gill mucosa contains a fully developed immune system, including commensal bacteria [77, 78]. This may indicate the important role of gill microbiota on fish survival. This assumption corresponds to our results, which showed gill microbial communities to change significantly, especially after infection in the non-UV treatments (Additional file 3: Table S7A), with high percentage of unique ASVs (Additional file 3: Table S7B). While previous research suggested microbial communities of different fish body sites significantly differ from one another [79, 80], Chiarello et al. [81] investigated both *Dicentrarchus labrax* and *S. aurata* fish species' different body parts (dorsal, anal, pectoral and caudal fins) showing significant differences in microbial communities . Rosado et al. [82] investigated the skin and gills of *D. labrax* and *S. aurata*, between December to February in healthy condition, and found that the different body sites of *D. labrax* hosted significantly different microbial communities. However, like our finding, *S. aurata* showed no significant differences between gills and body sites. Our results show that gill microbial communities, 24 h post infection (T_1_), differ from the other body sites, in agreement with our findings, it has been shown that fish reared in non-UV treated water showed better gill health in *Cyclopterus lumpus* fish [73]. Faith’s phylogenetic diversity (Fig. 2d) indeed shows non-UV treatment to have had a higher diversity indicating a more stable microbial community when compared to the UV treatment. To check whether these differences relate to the water microbiome, we generated an ordination plot including all samples for fish gills, lateral line, and abdomen area, for the first and second experiment (*S. aurata* infection experiments with *V. harveyi* in winter and summer season), highlighting the water samples (Additional file 1: Fig. S7). Water microbiome formed a different cluster and was statistically significant when compared to the fish microbiome. In addition, non-UV treated water also showed a higher diversity compared to UV treated water samples. These findings were previously reported by Chiarello et al. [81], who showed that microbial communities of different body parts of fish are different from water microbial communities, and other researchers showing UV treatment and membrane filtration to significantly reduce water microbial diversity [83, 84]. Chiarello et al. [81] showed that water samples have a similar diversity to different fish body parts, whereas our results showed that water samples have higher phylogenetic (PD) and Shannon diversity compared to the various fish body sites. This disagreement may relate to the experimental setup, seawater source, and body weight or fish age. Chiarello et al. [81] experimented on 7-year-old fish, while our experimental fish were younger (10 month to 1 year of age). However, a recent study that investigated *S. aurata* skin and gill microbial diversity at different ages and developmental stages showed age had no significant effect on the microbial communities [85]. In the same paper, and similar to our results, Rosado et al. [85], showed water alpha diversity to significantly differ from gills and skin of the *S. aurata* and had a higher Faith’s phylogenetic and Shannon diversity.

### Temporal changes in fish skin microbial community during health, disease, and recovery from bacterial infection

During the acute infection stage, the pathogenic bacteria significantly dominate fish skin microbial communities compared to the original microbial communities seen at T_0_ (pre-infection). During the recovery stage, the microbial communities were observed to gradually return to their initial microbial communities, which were present prior to infection (Figs. 4, 5, 6, 7). However, some variations were observed in microbial communities in relation to water treatment, season, fish type, the induced pathogens, and temporal resolutions. Zhang et al. [86] reported that *Ichthyophthirius multifiliis* infection affects bacterial symbiotic interaction as it decreases the abundance of teleost skin commensals and increases the colonization of opportunistic bacteria. Another recent paper investigated the effect of disease, antibiotic treatment, and recovery on the microbiome of *Dicentrarchus labrax* in the gills and skin. The results showed a significant decrease in fish skin microbial diversity following infection but an increased and different microbial diversity in the gills with asymmetry and unique community patterns [87]. Our results show (Fig. 2) the symmetrical decrease in microbial diversity indices of both fish gills and skin (abdomen and lateral line) following infection compared to T_0_, yet following infection at T_1_, gills had a higher microbial diversity compared to fish skin (Figs. 2, 4, 5). The increased microbial diversity of fish gills after infection observed by Rosado et al. [82] showed a different result than our finding (comparing T_0_ and T_1_). Interestingly, at T_2_ (1 week post infection) in the summer experiment, the microbial diversity of *S. aurata* gills increased compared to T_0_, but that was not the case in the winter experiments; this increase in microbial diversity was also seen on fish skin (Fig. 2). Rosado et al. [82] used different time scales for their sampling points during the “potential disease” stage, as the authors note, fish did not exhibit disease symptoms. In our sampling scheme, during the disease stage (24 h after infection; T_1_), fish clearly exhibited disease symptoms. Therefore, the disease stage indicated by Rosado et al. [82]may not correspond with ours, and that may explain some of the differences seen in gill microbial communities when comparing our results. Our results show that fish reared in UV treated water, a known water disinfectant, have a significantly lower microbial diversity compared to fish reared in non-UV water; we attributed the lower microbial diversity to the increase in fish mortality after infection of that group (Additional file 3: Table S5).

There were no significant variations in bacterial communities seen two weeks into the recovery stage, compared to the original state (T_0_). Researchers confirm that even if changes in the 16S bacterial composition are noticed during the recovery stage, the biochemical profile of the microbial community following disruptions goes back to its original state, highlighting that the original microbial composition may not be required in order to restore microbial original functions [28]. This can explain the non-significant differences in the microbial community composition seen in our results at the end of the recovery period, yet we did not perform microbial functional and biochemical pathway analyses. An interesting and important observation was seen in *S. aurata* at T_1_ (24 h post infection) when infected with *V. harveyi,* is the increased abundance of *Unclassified_Gammaproteobacteria* ASV in the summer 2015 experiment (Fig. 4). Furthermore, a remarkable increase of *Photobacterium (*belonging to the *Vibrio* family and closely related to the pathogenic *V. harveyi*) was observed in the repeated experiment in winter 2016 experiment (Fig. 5). Both the same *Unclassified_Vibrioceae* family ASVs and the *Unclassified_Gammaproteobacteria* ASV significantly dominated *S. aurata* skin during infection with *S. iniae* at the disease stage (Fig. 6). Moreover, these two families, *Unclassified_Vibrioceae* and at a lower percentage, the *Unclassified_Gammaproteobacteria* ASVs abundance were increased on fish skin during *S. iniae* infection in *L. calcarifer* (Fig. 7). These induced ASVs abundances were only noticed at T_1_ (24 h post infection) and at the disease stage (T_1–2_, T_1–3_), then they gradually decreased as fish progressed to the recovery stages (T_1–5_, T_2_ and T_3_; 5 days, one week and one month post infection, respectively). This could indicate the importance of these bacterial ASVs and their role in competing with pathogenic bacteria during infection. In this context, *Photobacterium* spp, members of *Vibrioceae* family are known for their symbiotic relation with different fish species [88]. Some are also considered opportunistic pathogens that adapt R-strategy and take advantage of the reduced fish microbial diversity during the infection and disease stages; when fish start to recover, these opportunistic pathogens' abundances decline [89, 90]. On the other hand, in our results, the *Unclassified_Gammaproteobacteria* class ASV showed a significantly elevated abundance during infection and disease stage (T_1_, Fig. 4), which may have played an important role in increased fish survival. Notice that fish subjected to UV treated water had lower survival rates, except in the third experiment (where *S. aurata* fish were infected with *s. iniae*), in which fish from the UV treatment showed a higher survival rate compared to the non-UV treatment. Interestingly, *Unclassified_Gammaproteobacteria* class ASV was highly abundant in that experiment at T_1_, on fish skin from the UV treatment compared to the non-UV treatment. A similar yet a different species, *Delftia* (Fig. 5) dominated *S. aurata* skin microbiota at T_0_; this species significantly declined during infection with *V. harveyi*. Yet, at T_1_ (24 h post infection) in the non-UV treatment we saw a higher abundance of *Delftia* ASV compared to the UV treatment, which may also be attributed to fish survival. Hence, a distinct ASV's following DeSeq analysis such as *Delftia, Rhodobacteraceae* and *firmicutes* were also found as a core microbiota in *S. aurata* [91, 92]*. Photobacterium* was also found to be higher after infection, this was also reported during mass mortalities of cultured *S. aurata* [93], which could be either as opportunistic pathogens or as a competing pathogen during *V. harveyi* infection in our case.

While there was no attempt to isolate those species nor perform a metabolic investigation during this study, our results indicate the importance of fish skin and gill microbiota in fish survival following infection. In addition, the results emphasize the need to preserve high bacterial diversity to mitigate fish pathogens, enhance fish health conditions and increase survival rates during infection.

## Conclusion

Various aspects of fish skin microbiome during healthy, diseased and recovery conditions were tested by a set of experiments that show how changes in innate and naturally occurring fish skin microbiome and dysbiosis affect fish health. We examined microbial diversity, composition, and survival rate of fish in UV and non-UV treated water before and after infection (with *Vibrio harveyi* and *Streptococcus iniae*) during the summer and winter seasons. The results demonstrate a higher survival rate of infected fish (*S. aurata* and *L. calcarifer*) in the non-UV treated water environment compared to UV-treated water. We noticed that the higher survival rate was attributed to a stable microbial community. When fish were subjected to UV treatment, the high resource supply per bacterium favored the fast-growing species (r-selection), therefore, different bacterial communities were significanlty changed when a strong pathogen was introduced and managed to dominate the fish microbiota. However, when the fish reacted to a less virulent pathogen (in case of *S. aurata* infected with *S. iniae*) K-selection strategies were maintained, resulting in lower mortality rates. This observation stresses the need to preserve high bacterial diversity to mitigate fish pathogens; bacterial diversity enhances fish health and increases survival during infection.

When examining the spatial variation in fish skin microbiome from different body sites of *S. aurata*, no significant variations were noticed. However, the microbial communities in gills significantly differentiated at specific time points, when compared with lateral lines site, before infection (T_0_) and 24 h post infection (T_1_). Gills host a high percentage of unique ASVs (up to 53% of the total microbial ASVs were unique for the gills), this may indicate that gill microbiota is key to fish survival.

Temporal changes in fish skin microbiome before and after infection, and throughout recovery, showed that microbial communities gradually restore to their initial pre-infection state. However, some variations were observed in the restored microbial communities; these may be related to water treatment, season, fish and pathogen species and temporal resolutions. Yet, temporal changes indicate the importance of certain bacterial species (ASVs) in disease development and fish survival rate, mainly *Delftia*, *Unclassified_Gammaproteobacteria* and *Unclassified_Vibrioceae* ASVs. The sequences of these bacterial ASV’s were patented under patent publication number WO2021038446. The potential to increase fish survival using these microbial species should be further investigated for future development of prophylaxis treatments to reduce the need for antibiotic application and reduce the adverse effects during bacterial infection outbreaks in aquaculture. Further research of metabolic pathways, functional diversity and bacterial isolation using similar experimental setups will increase understanding of disease ecology and shed light on important microbial functional traits and species that enhance fish survival.

## Supplementary Information

**Additional file 1.** Supplementry figures.

**Additional file 2.** Data Validation and inlet water physochemical analysis.

**Additional file 3.** Supplementary tables.

**Additional file 4.** Samples metadata file.

## Data Availability

Sequences used in this study were submitted to the National Center for Biotechnology Information (http://www.ncbi.nlm.nih.gov/) under Submission ID: SUB10986332 and BioProject ID: PRJNA800415.
