# Supplementary material for: Sparus aurata and Lates calcarifer skin microbiota under healthy and diseased conditions in UV and non-UV treated water
Source: Anim Microbiome. 2022 Jun 21;4:42. doi: 10.1186/s42523-022-00191-y (PMC9210813; doi:10.1186/s42523-022-00191-y)
Supplement: Supplementary file 1 — Additional file 1. Supplementry figures. [file 42523_2022_191_MOESM1_ESM.docx]

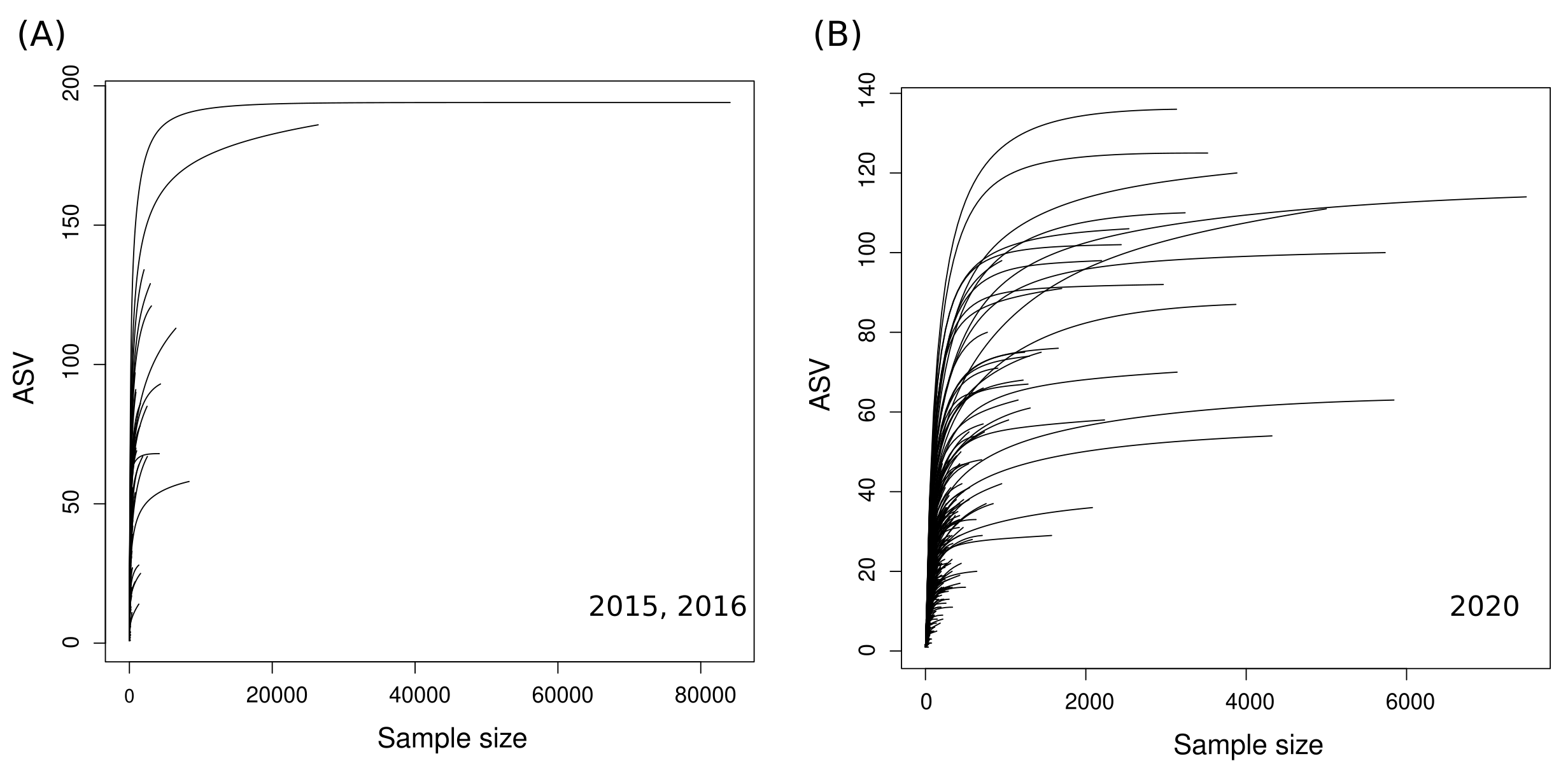
**Supplementary Figure S1.** Rarefaction curves for all 2015, 2016 (A) and 2020 (B) collected samples.


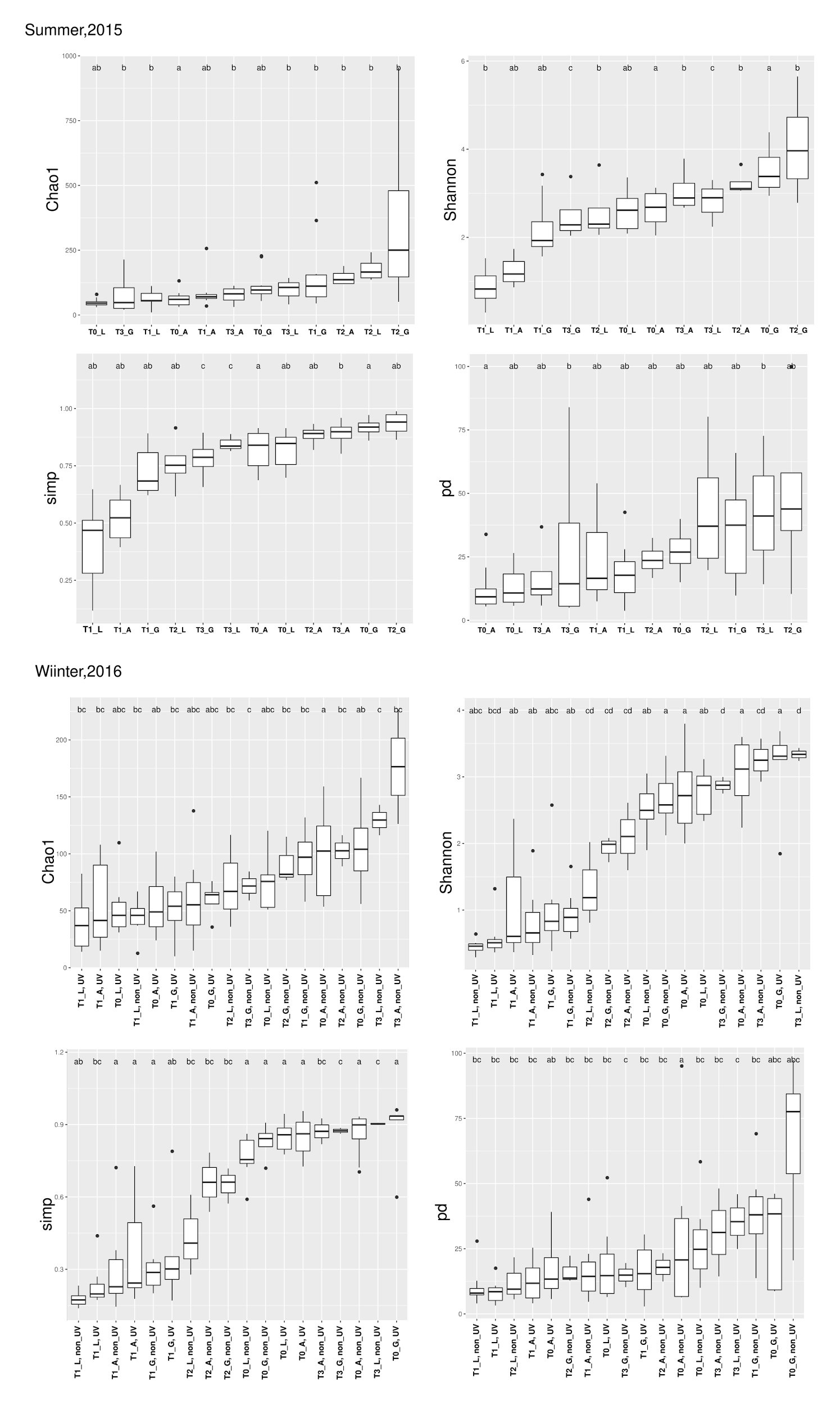


**Supplementary Figure S2.** Tukey’s test of significance for different diversity indices between different time points and body sites during *V. harveyi* infection in summer, 2015 and winter 2016.


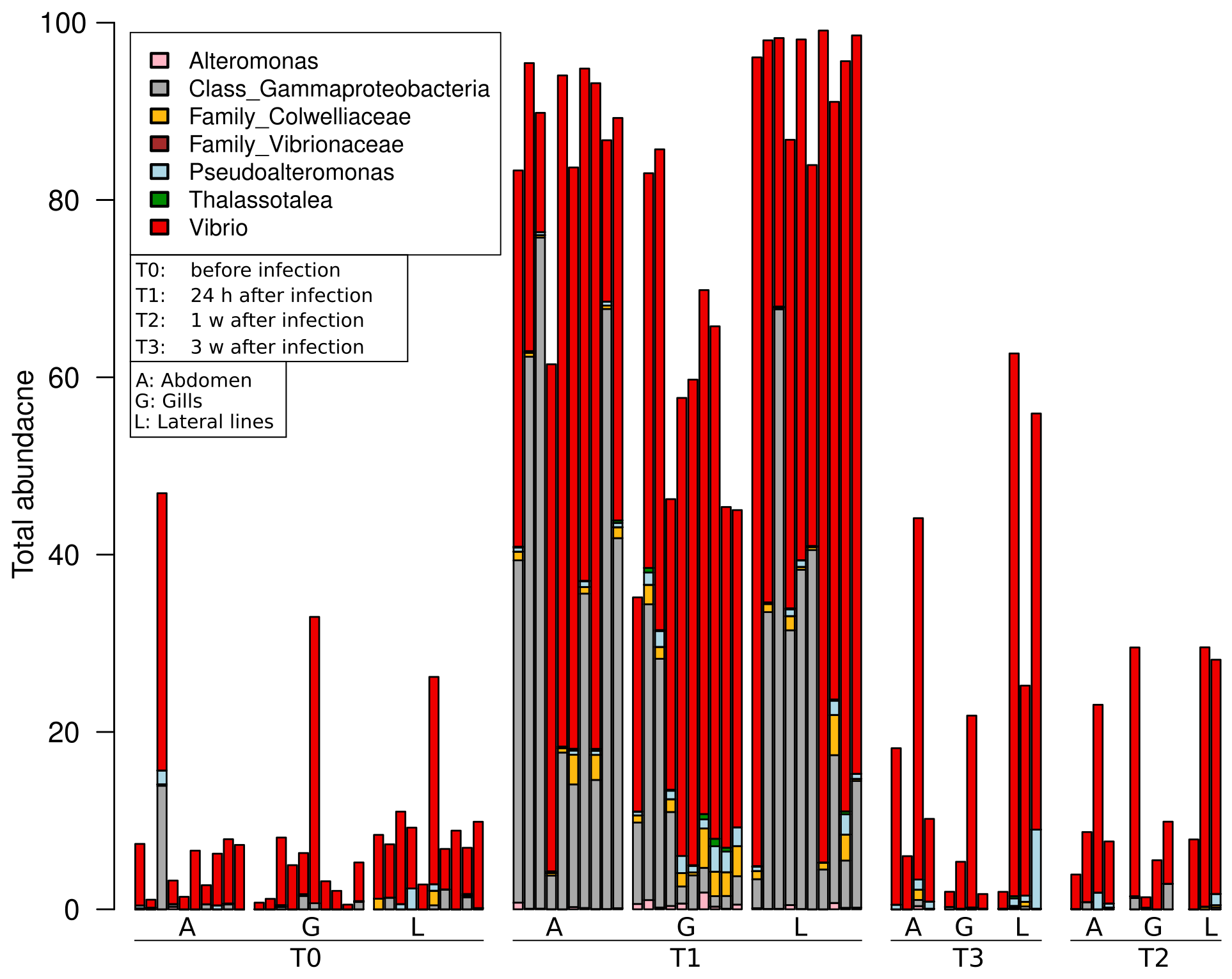


**Supplementary Figure S3.** Bar graph illustrating DeSeq analysis showing relative abundances of differential significant ASV’s (different colors). Each bar represents one (tagged) fish at different time points (T_0_ before infection, T_1_ 24h, T_2_ one week, T_3_ three weeks post infection), body sites (Abdomen (A); Gills (G); Lateral line (L)) for non-UV treatment during *V. harveyi* infection in summer, 2015.


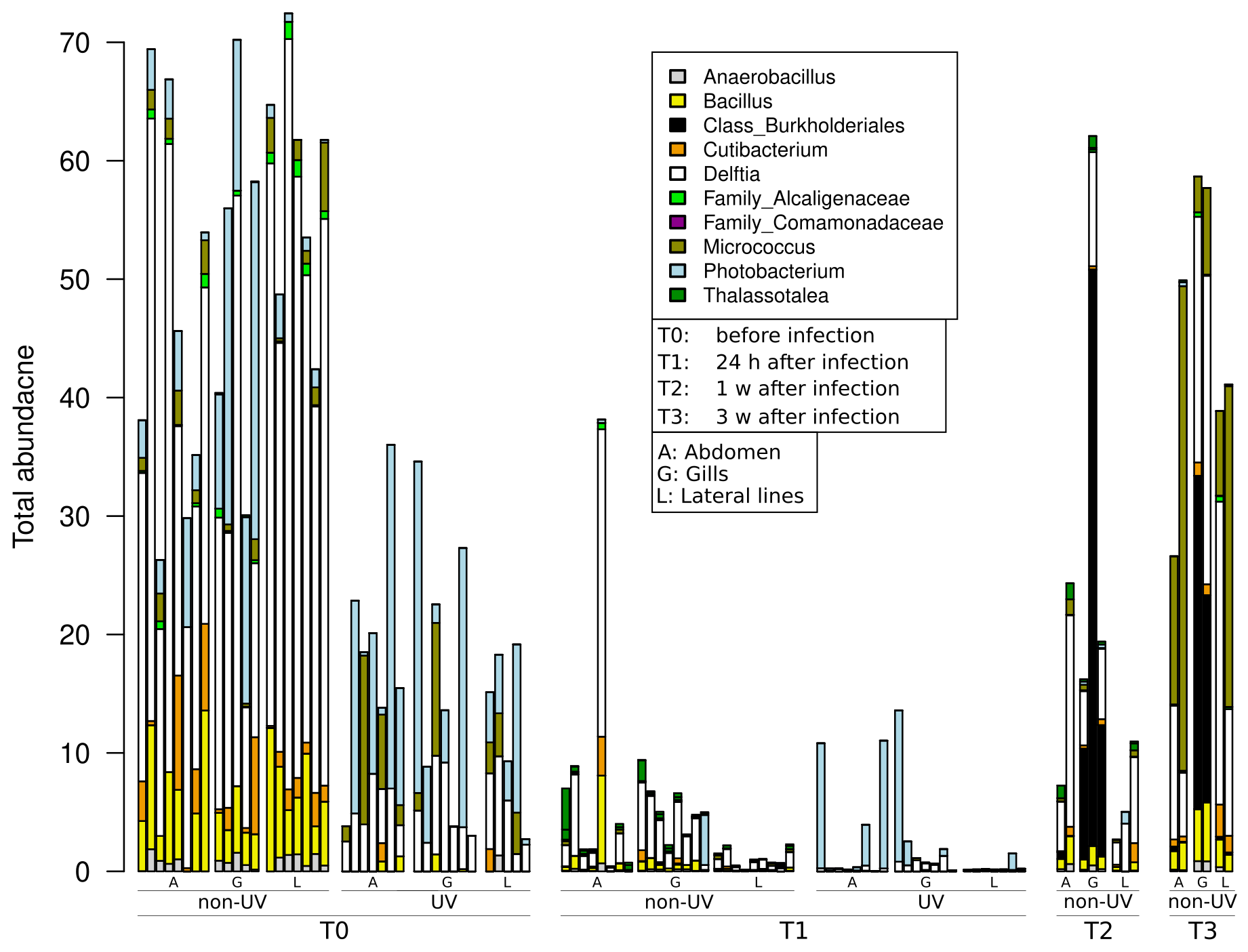


**Supplementary Figure S4.** Bar graph illustrating DeSeq analysis showing relative abundances of differential significant ASV’s (different colors). Each bar represents one (tagged) fish at different time points (T_0_ before infection, T_1_ 24h, T2 one week, T_3_ three weeks post infection), different body sites (Abdomen (A); Gills (G); Lateral line (L)) and at different UV treatments during *V. harveyi* infection in winter, 2016.


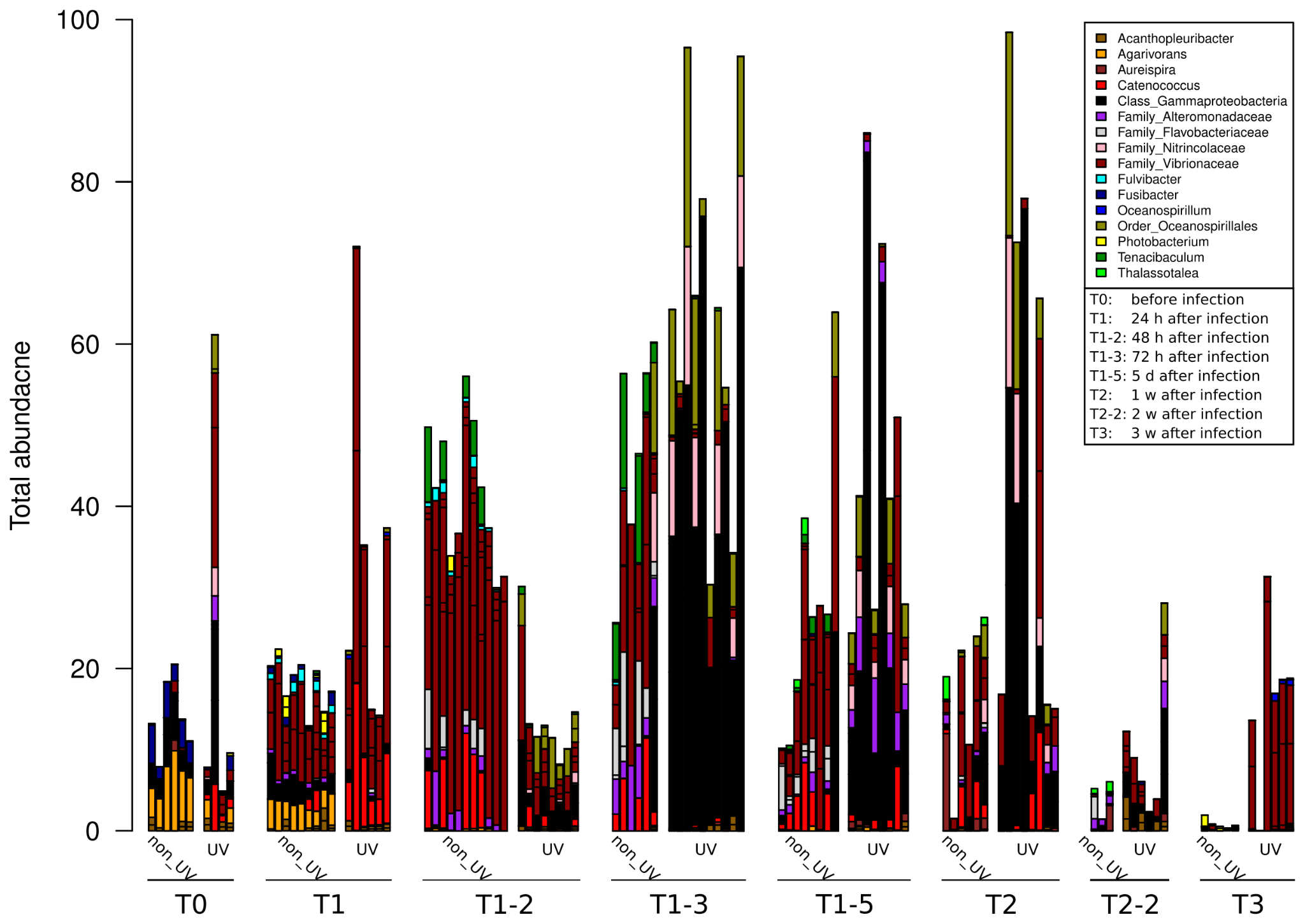


**Supplementary Figure S5.** Bar graph illustrating DeSeq analysis showing relative abundances of differential significant ASV’s (different colors). Each bar represents one (tagged) fish at different time points (T_0_ before infection, T_1_ 24 h, T_1-2_ 48 h, T_1-3_ 72 h, T_1-5_ five days, T_2_ one week, T_2-2_ two weeks and T_3_ three weeks post infection), for UV and non-UV treatments during *S. iniae* infection in *S. aurata.*


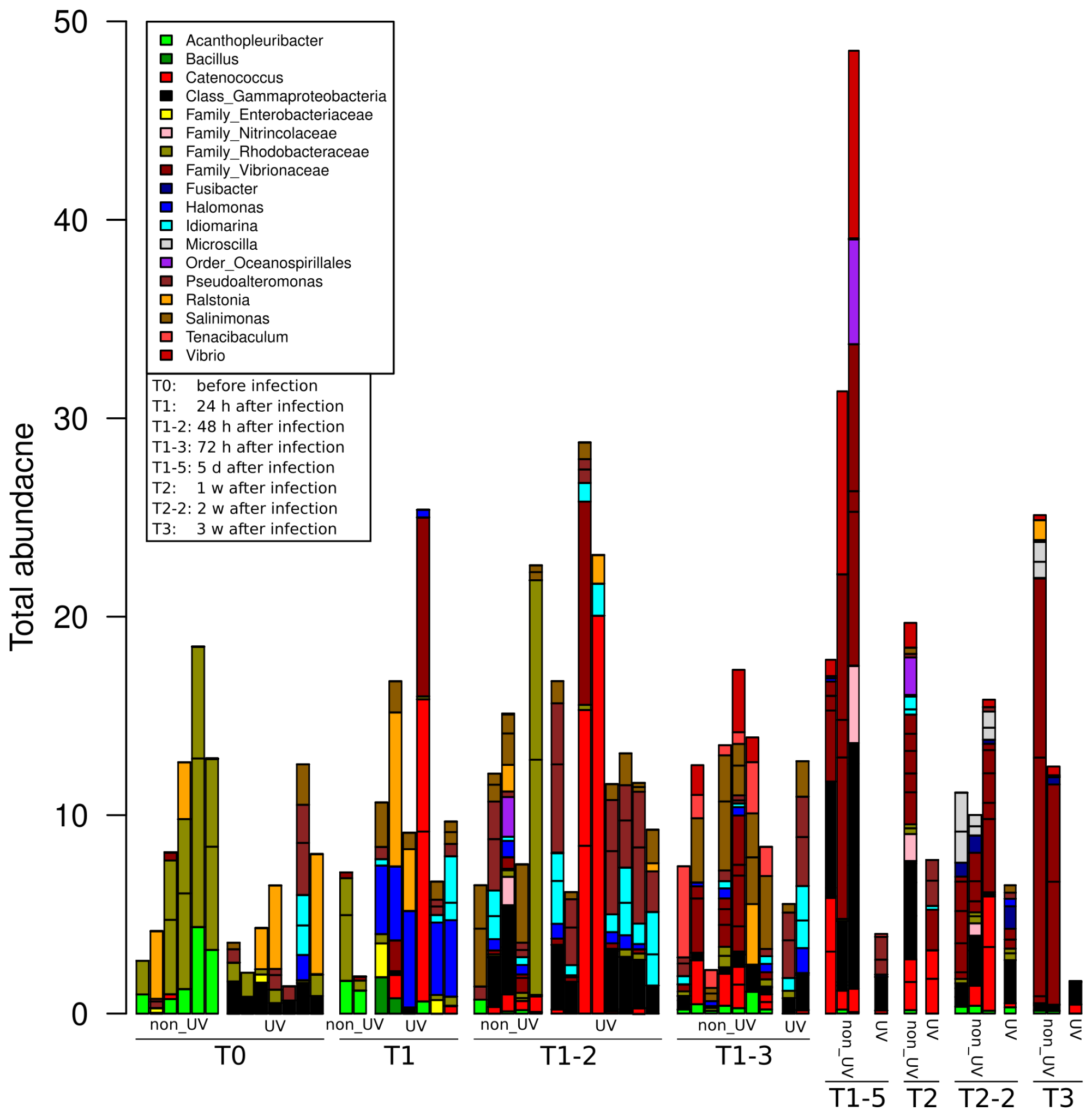
**Supplementary Figure S6.** Bar graph illustrating DeSeq analysis showing relative abundances of differential significant ASV’s (different colors). Each bar represents one (tagged) fish at different time points (T_0_ before infection, T_1_ 24 h, T_1-2_ 48 h, T_1-3_ 72 h, T_1-5_ five days, T_2_ one week, T_2-2_ two weeks and T_3_ three weeks post infection), for UV and non-UV treatments during *S. iniae* infection in *L. calcarifer*.


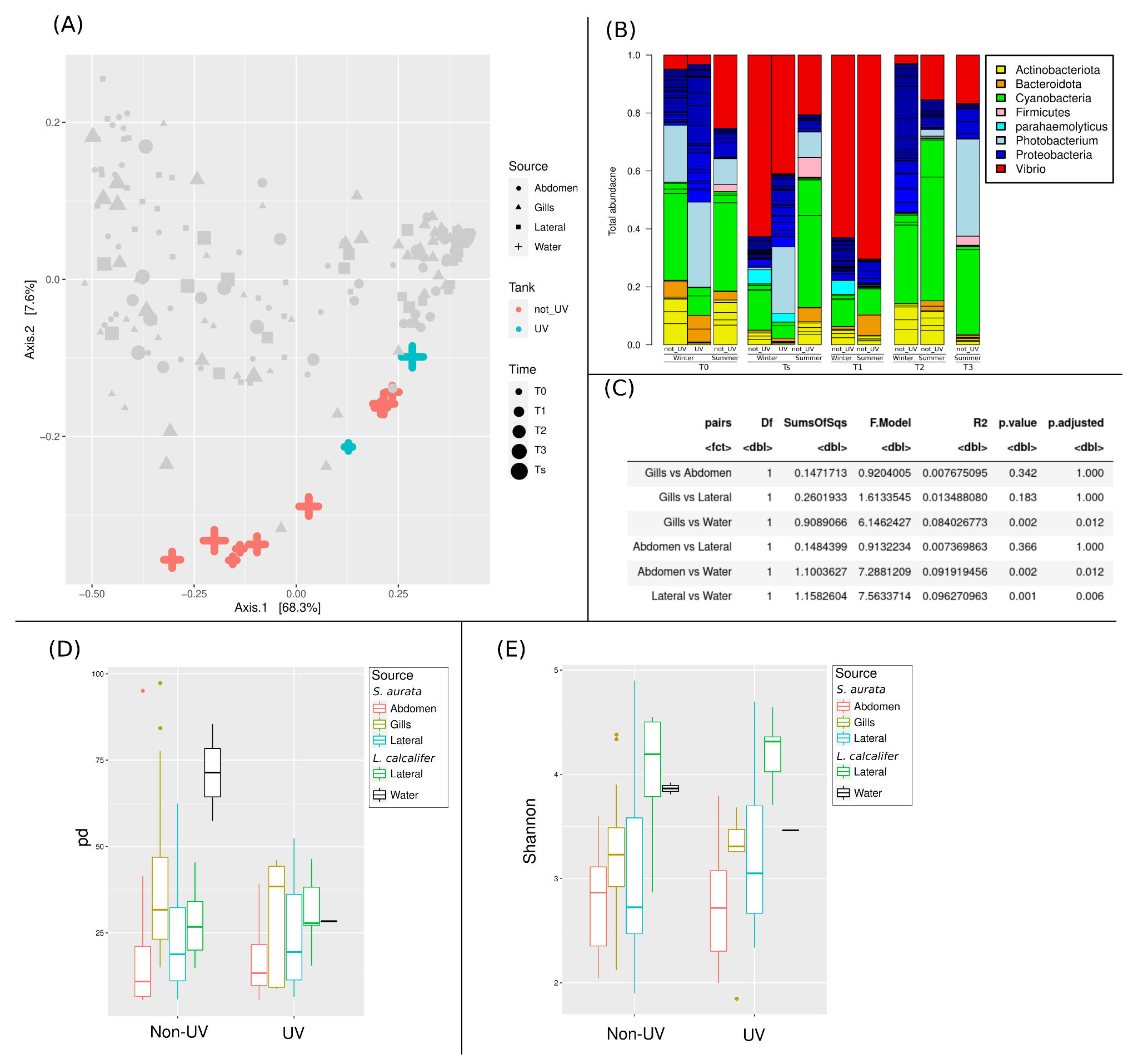


**Supplementary Figure S7.** PCoA for all taken samples (gray) highlighting only water samples (A), bar graph showing the relative bacterial abundance for different water samples taken at the summer and winter seasons of 2015 and 2016 for UV and non-UV tank (B). (C) showing a statistical pairwise significance between water samples and different fish body sites and (D) a phylogenetic (pd) and (E) Shannon diversity indices for all fish experiments grouped at T_0_ before infection for non-UV and UV treated water tanks.


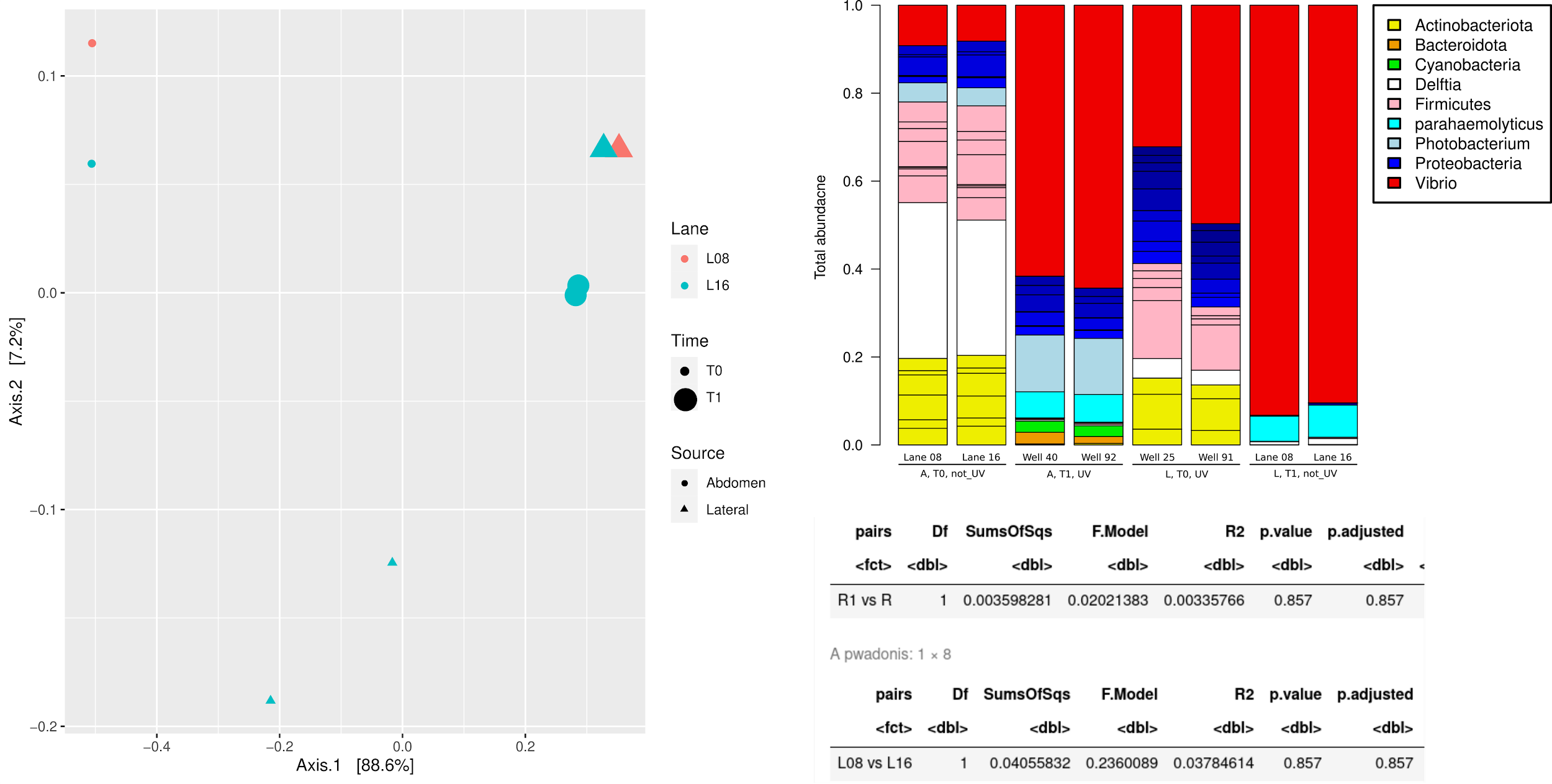


**Supplementary Figure S8.** PCoA, bar graph and statistical analysis of similarity (adonis) showing no significant difference between the replicate samples when placed in different MiSeq lanes nor was there a significant batch effect within the same MiSeq lane
