# Supplementary material for: Sparus aurata and Lates calcarifer skin microbiota under healthy and diseased conditions in UV and non-UV treated water
Source: Anim Microbiome. 2022 Jun 21;4:42. doi: 10.1186/s42523-022-00191-y (PMC9210813; doi:10.1186/s42523-022-00191-y)
Supplement: Supplementary file 2 — Additional file 2. Data Validation and inlet water physochemical analysis. [file 42523_2022_191_MOESM2_ESM.docx]

Data validation:

In *S. aurata* and during *V. harveyi* infection experiments (the first and second experiments corresponding to summer, 2015 and winter, 2016; respectively) we have investigated the effect of different variable region amplification of 16S rDNA on the obtained fish skin microbial diversity in UV and non-UV treated water. Therefore, different primers were used (**Supplementary** **Table 1**) to assess bacterial diversity and community composition of fish skin microbiome. The total number of collected samples from first and second experiments were 183 samples which ran in 3 lanes of Illumina MiSeq 150 PE reads, in addition a total of 5 samples were added as negative control (2 samples as “PCR negative” and 3 samples as “Illumina negative”), 4 replica samples (for different lanes and within lane replica) and 9 water samples (total 201 samples) were distributed among and within different runs (**Supplementary File SF1**). The average sequence number per each primer set varied significantly for the different regions of amplification (**Supplementary Table 1**). Sequenced data showed the third primer set amplifying the V4 region of 16S rDNA (F649 and R889) was able to obtain the highest number of raw sequences with an average raw sequence number of 48,791 ± 47,472. This primer set retained its rank amongst all other primer sets even after the quality control (QC) procedure, with 43,510 ± 44,025 high-quality sequences. This quality control step included removal of low quality and incomplete sequences, removal of chimeric sequences and discarding of chloroplasts, mitochondrial and unclassified bacterial sequences (**Supplementary Table 1**). Thus, we based all further analysis on the V4 region of 16S rRNA (F649-R889 primer set), therefore, and for the third and fourth experiment during *S. iniae* infection experiments in 2020, we used the universal 16S V4 region primer (F515 and R806, **Supplementary Table S3**). Different replicas between different lanes and within the same lane for batch effect were also analyzed and showed no significant difference (**Supplementary Figure S8).** The negative controls (DNA extraction, PCR, sequencing negative controls) retained a low sequence number and were filtered out in the first step of removing samples containing less than 1000 ASV.

Used *V. harveyi* sequence for infecting fish experiment

*>TNNNANCTGGCGGCAGGCCTAACACATGCAAGTCGAGCGGAAACGAGTTATCTGAACCTTCGGGGGACGATAACGGCGTCGAGCGGCGGACGGGTGAGTAATGCCTAGGAAATTGCCCTGGTGTGGGGGATAACCATTGGAAACGATGGCTAATACCGCATAATACCTACGGGTCAAAGAGGGGGACCTTCGGGCCTCTCGCGTCAGGATATGCCTAGGTGGGATTAGCTAGTTGGTGAGGTAATGGCTCACCAAGGCGACGATCCCTAGCTGGTCTGAGAGGATGATCAGCCACACTGGAACTGAGACACGGTCCAGACTCCTACGGGAGGCAGCAGTGGGGAATATTGCACAATGGGCGCAAGCCTGATGCAGCCATGCCGCGTGTGTGAAGAAGGCCTTCGGGTTGTAAAGCACTTTCAGTCGTGAGGAAGGTAGTGTAGTTAATAGCTGCATTATTTGACGTTAGCGACAGAAGAAGCACCGGCTAACTCCGTGCCAGCAGCCGCGGTAATACGGAGGGTGCGAGCGTTAATCGGAATTACTGGGCGTAAAGCGCATGCAGGTGGTTTGTTAAGTCAGATGTGAAAGCCCGGGGCTCAACCTCGGAATAGCATTTGAAACTGGCAGACTAGAGTACTGTAGAGGGGGGTAGAATTTCAGGTGTAGCGGTGAAATGCGTAGAGATCTGAAGGAATACCGGTGGCGAAGGCGGCCCCCTGGACAGATACTGACACTCAGATGCGAAAGCGTGGGGAGCAAACAGGATTAGATACCCTGGTAGTCCACGCCGTAAACGATGTCTACTTGGAGGTTGTGGCCTTGAGCCGTGGCTTTCGGAGCTAACGCGTTAAGTAGACCGCCTGGGGAGTACGGTCGCAAGATTAAAACTCAAATGAATTGACGGGGGCCCGCACAAGCGGTGGAGCATGTGGTTTAATTCGATGCAACGCGAAGAACCTTACCTACTCTTGACATCCAGAGAACTTTCCAGAGATGGATTGGTGCCTTCGGGAACTCTGAGACAGGTGCTGCATGGCTGTCGTCAGCTCGTGTTGTGAAATGTTGGGTTAAGTCCCGCAACGAGCGCAACCCNTATCCTNGTTTGCCAGCACTTCCGGTGGGAACTCCAAGGAGACTGCCCGGTGATAAACCCGGAAGGNNNT*

Used *V. harveyi* sequence for infecting fish experiment

*>CNNNNNNGNCTGNCNNCNTGCCTAATACATGCTAGTAGAACGCTGACGCATTGGTGCTTGCACTAATCCAAAGAGTTGCGAACGGGTGAGTAACGCGTAGGTAACCTACCTCATAGCGGGGGATAACTATTGGAAACGATAGCTAATACCGCATGACACTAGAGTACACATGTACTTAATTTAAAAGGAGCAAATGCTTCACTATGAGATGGACCTGCGTTGTATTAGCTAGTTGGTGAGGTAACGGCTCACCAAGGCGACGATACATAGCCGACCTGAGAGGGTGATCGGCCACACTGGGACTGAGACACGGCCCANACTCCTACGGGAGGCAGCAGTAGGGAATCTTCGGCAATGGACGGAAGTCTGACCGAGCAACGCCGCGTGAGTGAAGAAGGTTTTCGGATCGTAAAGCTCTGTTGTTAGAGAAGAACGGTAATGGGAGTGGAAAATCCATTACGTGACGGTAACTAACCAGAAAGGGACGGCTAACTACGTGCCAGCAGCCGCGGTAATACGTATGTCTCGAGCGTTGTCCGGATTTATTGGGCGTAAAGCGAGCGCAGGCGGTTCTATAAGTCTGATATAATAGGCAGTGGCTCAACCATTGTATGCTTTGGANACTGTAGAACTTGAGTGCAGAAGGGGAGAGTGGAATTCCATGTGTACCGGTGAAATGCGTATATATATGGNGGAACACCGGTGGCGAAAACGGCTGTCTGCTCTGTATCTGACGCTGCNGCTCGAAAGCGTGGAGAGCNAANAGCATTATACACCCTGGTTGTCCCNCNATNNACNACGAGTGCTACGTGTNANGGGGTTTNCGCNGCNTANTGNNGCGCTNACATATTAACTCTCCGCNTGNAGNGACGANCNCNCGGNTGNNNTCNNNNANTNNNNNGCNCNCCCCCCNNTNTCCNNGTAATNNANNCNNACTACGNNCANANNTNNNANNNCGTGNNGNNNGTCGNTNNNNNCNNNNTATNTNTGTNNNNNCACNNNNNANAANGNNATNCACNNTNNCNTNNNCT*

Table 1 Sea water physical, chemical, and biological analysis during experimentation in 2015, 2016 and 2020

| Parameters | 2015, Sep | 2016, Jan | 2020, Feb | 2020, Mar |
| --- | --- | --- | --- | --- |
| pH | 8.55 | 8.52 | 8.44 | 8.47 |
| BOD [mg/l] | 0.5 | 1.1 | 0.2 | 0.5 |
| TSS [mg/l] | 1.2 | 0.9 | 0.3 | 0.4 |
| TOC [mg/l] | 1.25 | 0.91 | 1.01 | 0.85 |
| Turbidity (NTU) | 1.5 | 0.8 | 0.52 | 0.1 |
| NH4-N [mg/l] | 0.01 | 0.01 | 0.01 | 0.01 |
| (NO2+NO3)-N [mg/l] | 0.01 | 0.01 | 0.01 | 0.02 |
| TN [mg/l] | 0.01 | 0.17 | 0.07 | 0.13 |
| PO4-P [mg/l] | 0.01 | 0.01 | 0.01 | 0.01 |
| TP [mg/l] | 0.01 | 0.01 | 0.18 | 0.01 |
| FC [1/100 ml] | 0 | 0 | 0 | 0 |
| Cu [mg/l] | 0.05 | 0.05 | 0.05 | 0.05 |
