# Supplementary material for: Sparus aurata and Lates calcarifer skin microbiota under healthy and diseased conditions in UV and non-UV treated water
Source: Anim Microbiome. 2022 Jun 21;4:42. doi: 10.1186/s42523-022-00191-y (PMC9210813; doi:10.1186/s42523-022-00191-y)
Supplement: Supplementary file 3 — Additional file 3. Supplementary tables. [file 42523_2022_191_MOESM3_ESM.docx]

**Supplementary Table 1.** The PCR I 16S rDNA primer sets for the *V. harveyi* infection experiments in summer 2015 and winter 2016 (sequences grouped together) showing the average ± SD numbers of raw and classified sequences and sample number (n) taken from Fuks et al. (2018).

| Forward primer (3`-5`) [Conc] | Reverse primer (3`-5`) [Conc] | Raw sequences ± SD | Classified sequences (number of samples) |
| --- | --- | --- | --- |
| F70:TGGCGAACGGGTGAGTAA [10 µM] | R307:GACGTGTGCTCTTCCGATCTCCGTGTCTCAGTCCCARTG [20 µM] | 16,894 ± 26,072 | 16,783 ± 25,651 (n=173) |
| F310:ACTCCTACGGGAGGCAGC [10 µM] | R499:AGACGTGTGCTCTTCCGATCTGTATTACCGCGGCTGCTG [10 µM] | 4,312 ± 5,387 | 4,282 ± 5,383 (n=57) |
| F649:GTGTAGCGGTGRAATGCG [20 µM] | R889:AGACGTGTGCTCTTCCGATCTCCCGTCAATTCMTTTGAGTT [20 µM] | 45,464 ± 43,892 | 44,920 ± 43,374 (n=194) |
| F908:GGAGCATGTGGWTTAATTCGA [20 µM] | R1067:AGACGTGTGCTCTTCCGATCTCGTTGCGGGACTTAACCC [10 µM] | 19,999 ± 21,659 | 20,042 ± 21,611 (n=115) |
| F1139:GGAGGAAGGTGGGGATGAC [10 µM] | R1364:AGACGTGTGCTCTTCCGATCTAAGGCCCGGGAACGTATT [10 µM] | 6,307 ± 11,315 | 6,668 ± 11,820 (n=9) |

**Supplementary Table 2.** PCR II primers sequences and working concentrations for the *V. harveyi* infection experiment.

| Primer | Sequence (5`-3`) [Concentration] |
| --- | --- |
| F70 | AATGATACGGCGACCACCGAGATCTACACTCTTTCCCTACACGACGCTCTTCCGATCTTGGCGAACGGGTGAGTAA [4 µM] |
| F310 | AATGATACGGCGACCACCGAGATCTACACTCTTTCCCTACACGACGCTCTTCCGATCTACTCCTACGGGAGGCAGC [4 µM] |
| F649 | AATGATACGGCGACCACCGAGATCTACACTCTTTCCCTACACGACGCTCTTCCGATCTGTGTAGCGGTGRAATGCG [8 µM] |
| F908 | AATGATACGGCGACCACCGAGATCTACACTCTTTCCCTACACGACGCTCTTCCGATCTGGAGCATGTGGWTTAATTCGA [8 µM] |
| F1139 | AATGATACGGCGACCACCGAGATCTACACTCTTTCCCTACACGACGCTCTTCCGATCTGGAGGAAGGTGGGGATGAC [4 µM] |
| Reverse primer | CAAGCAGAAGACGGCATACGAGAT*NNNNNNNN*GTGACTGGAGTTCAGACGTGTGCTCTTCCGATCT [8 µM] |

**Supplementary Table S3.** The PCR I and II 16S rDNA primer sets [(Caporaso et al. 2011)](https://sciwheel.com/work/citation?ids=185575&pre=&suf=&sa=0) for the *S. iniae* infection experiment.

| PCR | Forward primer (5`-3`) [Conc] | Reverse primer (5`-3`) [Conc] |
| --- | --- | --- |
| PCR I | PCRI-F515:TCGTCGGCAGCGTCAGATGTGTATAAGAGACAGGTGYCAGCMGCCGCGGTAA[4 µM] | PCRI-R806:GTCTCGTGGGCTCGGAGATGTGTATAAGAGACAGGACTACNVGGGTWTCTAAT [4 µM] |
| PCR II | PCRII-F515:AATGATACGGCGACCACCGAGATCTACACTCTTTCCCTACACGACGCTCTTCCGATCTGTGTAGCGGTGRAATGCG [8 µM] | Reverse-primer:CAAGCAGAAGACGGCATACGAGAT*NNNNNNNN*GTGACTGGAGTTCAGACGTGTGCTCTTCCGATCT [8 µM] |

###

**Supplementary Table S4.** Average and standard deviations of obtained ASV’s and after data curation

| Dataset | ASV sequences (Avg. ± SD) | Curated sequences (Avg. ± SD) |
| --- | --- | --- |
| *V. harveyi* infection experiment | 45,464 ±43,893 | 44,920 ± 43,375 |
| *S. iniae* infection experiment | 30,464 ± 22,104 | 30,882 ± 23,982 |

**Supplementary Table S5:** UV vs non-UV treatments, adonis-pairwise significance test for different body sites at different time points in all infectious experiments.

|  | **Non-UV** | | |
| --- | --- | --- | --- |
|  | *S. aurata* | | *L. calcarifer* |
|  | *V. harveyi* | *S.iniae* | *S.iniae* |
| **UV** | **A-T0 = 0.004** G-T0 = 0.118 **L-T0 = 0.001** ---------------- A-T1 = 0.254 G-T1 = 0.366 L-T1 = 0.170 | **L-T0 = 0.008 L-T1 = 0.002 L-T1-2 = 0.001 L-T1-3 = 0.001 L-T1-5 = 0.001 L-T2 = 0.017 L-T2-2 = 0.015 L-T3 = 0.003** | **L-T0 = 0.048**  L-T1 = 0.539 L-T1-2 = 0.059 L-T1-3 = 0.383 L-T1-5 = 0.250 L-T2 = 0.667 L-T2-2 = 0.250 L-T3 = 0.333 |

**Supplementary Table S6.** Absolute and weighted percentages of unique and shared ASV’s for UV vs non-UV treatments at different time points and body sites, during *V. harveyi* (A) and *S. iniae* (B) infection.

(A)

| **Time** | **Body sites** | **Percent** | | | **Weighted** | | |
| --- | --- | --- | --- | --- | --- | --- | --- |
|  |  | **Non-UV** | **UV** | **Shared** | **Non-UV** | **UV** | **Shared** |
| **T0** | **Abdomen** | 45% | 31% | 16% | 14% | 8% | 77% |
|  | **Gills** | 59% | 29% | 12% | 13% | 17% | 69% |
|  | **Lateral** | 49% | 34% | 17% | 10% | 11% | 79% |
| **T1** | **Abdomen** | 41% | 40% | 19% | 1% | 3% | 96% |
|  | **Gills** | 56% | 24% | 20% | 2% | 1% | 97% |
|  | **Lateral** | 40% | 41% | 19% | 1% | 2% | 98% |
| **T0, T1 and T2** | **All** | **58%** | **20%** | **21%** | **5%** | **2%** | **93%** |

(B)

| **Time** | ***S. aurata*** | | | | | | ***L. calcarifer*** | | | | | |
| --- | --- | --- | --- | --- | --- | --- | --- | --- | --- | --- | --- | --- |
|  | **Percent** | | | **Weighted** | | | **Percent** | | | **Weighted** | | |
|  | **Non-UV** | **UV** | **shared** | **Non-UV** | **UV** | **shared** | **Non-UV** | **UV** | **shared** | **Non-UV** | **UV** | **shared** |
| **T0** | 44% | 29% | 26% | 11% | 11% | 79% | 35% | 46% | 20% | 17% | 14% | 70% |
| **T1** | 39% | 40% | 22% | 11% | 9% | 80% | 18% | 69% | 13% | 14% | 22% | 64% |
| **T1-2** | 40% | 41 | 19% | 6% | 17% | 76% | 48% | 29% | 23% | 16% | 3% | 84% |
| **T1-3** | 40% | 36% | 23% | 7% | 5% | 88% | 54% | 24% | 21% | 14% | 6% | 80% |
| **T1-5** | 44% | 38% | 18% | 14% | 10% | 76% | 63% | 26% | 11% | 33% | 20% | 47% |
| **T2** | 45% | 29% | 26% | 10% | 6% | 84% | 67% | 24% | 9% | 35% | 21% | 44% |
| **T2-2** | 32% | 43% | 25% | 10% | 13% | 77% | 59% | 24% | 17% | 30% | 15% | 54% |
| **T3** | 49% | 30% | 21% | 18% | 18% | 64% | 70% | 23% | 7% | 43% | 32% | 25% |
| **All time points** | **41%** | **27%** | **31%** | **4%** | **3%** | **93%** | **45%** | **30%** | **25%** | **11%** | **4%** | **85%** |

**Supplementary Table S7.** Body sites adonis-pairwise significance test at different time points for *S. aurata* during *V. harveyi* infection at summer seasons (only non-UV treatments) and winter (non-UV and UV treatments) (A), and unique unweighted and weighted ASV’s at different body sites in the non-UV treatment both summer and winter experiments (B).

(A)

| **Season** | **Summer 2015** | | **Winter 2016** | | | |
| --- | --- | --- | --- | --- | --- | --- |
| **Treatment** | **Non-UV** | | **Non-UV** | | **UV** | |
| **Body site** | **Abdomen** | **Gills** | **Abdomen** | **Gills** | **Abdomen** | **Gills** |
| **Gills** | T0 = 0.228 **T1 = 0.018** T2 = 1.000 T3 = 1.000 | - | T0 = 0.084 T1 = 1.000 T2 = 1.000 T3 = 1.000 | - | T0 = 1.000 T1 = 1.000 | - |
| **Lateral** | T0 = 1.000 T1 = 0.492 T2 = 0.750 T3 = 1.000 | **T0 = 0.006 T1 = 0.012** T2 = 0.162 T3 = 1.000 | T0 = 0.348 T1 = 0.090 T2 = 1.000 T3 = 1.000 | **T0 = 0.012 T1 = 0.006** T2 = 0.600 T3 = 1.000 | T0 = 1.000 T1 = 1.000 | T0 = 1.000 T1 = 0.510 |

(B)

| **Season** | **Summer 2015** | | **Winter 2016** | |
| --- | --- | --- | --- | --- |
| **Treatment** | **Non-UV** | | **Non-UV** | |
| **Body site** | **Unweighted** | **Weighted** | **Unweighted** | **Weighted** |
| **Abdomen** | T0 = 18%  **T1 = 15%**  T2 = 13%  T3 = 23% | T0 = 4%  **T1 = <1%**  T2 = 4%  T3 = 4% | T0 = 28%  T1 = 15%  T2 = 24%  T3 = 39% | T0 = 4%  T1 = 1%  T2 = 2%  T3 = 6% |
| **Gills** | **T0 = 49%**  **T1 = 53%**  T2 = 53%  T3 = 26% | **T0 = 9%**  **T1 = 2%**  T2 = 8%  T3 = 3% | **T0 = 28%**  **T1 = 52%**  T2 = 27%  T3 = 11% | **T0 = 4%**  **T1 = <1%**  T2 = 2%  T3 = 3% |
| **Lateral** | **T0 = 12%**  **T1 = 8%**  T2 = 16%  T3 = 22% | **T0 = <1%**  **T1 = 8%**  T2 = 3%  T3 = 4% | **T0 = 20%**  **T1 = 7%**  T2 = 25%  T3 = 27% | **T0 = 4%**  **T1 = <1%**  T2 = 1%  T3 = 7% |

**Supplementary Table S8** adonis-pairwise significance test at different time points for *S. aurata* during *V. harveyi* infection at winter and summer seasons in non-UV and UV treatment (A), for *S. aurata* during *S. iniae* infection at non-UV (B), UV treatment (C), and for *L. clarifier* during *S. iniae* infection at non-UV (D), UV treatment (E).

| **(A) Pairwise comparisons for UV and non-UV treatments for *S. aurata* during *V. harveyi* infection at winter and summer seasons** | | | | | | | |
| --- | --- | --- | --- | --- | --- | --- | --- |
| **Season** | **Summer 2015** | | | **Winter 2016** | | | |
|  | **Non-UV** | | | **Non-UV** | | | **UV** |
|  | **T0** | **T1** | **T2** | **T0** | **T1** | **T2** | **T0** |
| **T1** | **A = 0.012 G = 0.006 L = 0.006** | - |  | **A = 0.006 G = 0.012 L = 0.006** | - |  | **A = 0.001 G = 0.003 L = 0.001** |
| **T2** | A = 0.924 G = 1.000 **L = 0.018** | **A = 0.006 G = 0.012 L = 0.006** | - | A = 0.144 G = 0.942 **L = 0.024** | A = 0.450 **G = 0.048 L = 0.036** | - | - |
| **T3** | A = 1.000 G = 0.702 L = 0.084 | **A = 0.006 G = 0.018 L = 0.018** | A = 1.000 G = 1.000 L = 0.378 | A = 0.276 G = 0.252 L = 0.186 | A = 0.168 G = 0.222 L = 0.150 | A = 1.000 G = 1.000 L = 0.600 | - |

| **(B) Non-UV treatment pairwise comparisons for *S. aurata* during *S. iniae* infection at winter seasons 2020** | | | | | | | | |
| --- | --- | --- | --- | --- | --- | --- | --- | --- |
| **Time** | **T0** | **T1** | **T1-2** | **T1-3** | **T1-5** | **T2** | **T2-2** | **T3** |
| **T1** | **0.005** | - | - | - | - | - | - | - |
| **T1-2** | **0.002** | **<0.001** | - | - |  |  |  | - |
| **T1-3** | 0.056 | **0.007** | 0.056 | - | - | - | - | - |
| **T1-5** | **0.009** | **0.001** | **<0.001** | 0.168 | - | - | - | - |
| **T2** | 0.064 | **0.003** | **0.002** | 0.112 | **0.009** | - | - | - |
| **T2-2** | 0.341 | 0.168 | 0.056 | 0.532 | 0.166 | 0.364 | - | - |
| **T3** | 0.056 | **0.013** | **0.006** | 0.112 | **0.023** | 0.056 | 0.672 | - |

| **(C) UV treatment pairwise comparisons for *S. aurata* during *S. iniae* infection at winter seasons 2020** | | | | | | | | |
| --- | --- | --- | --- | --- | --- | --- | --- | --- |
| **Time** | **T0** | **T1** | **T1-2** | **T1-3** | **T1-5** | **T2** | **T2-2** | **T3** |
| **T1** | 1.000 | - | - | - | - | - | - | - |
| **T1-2** | 0.168 | **0.009** | - | - |  |  |  | - |
| **T1-3** | 0.084 | **0.002** | **0.002** | - | - | - | - | - |
| **T1-5** | 0.504 | **0.028** | **0.004** | 0.362 | - | - | - | - |
| **T2** | 0.616 | 0.112 | **0.004** | 1.000 | 1.000 | - | - | - |
| **T2-2** | 0.896 | 0.084 | **0.007** | **0.003** | **0.031** | 0.700 | - | - |
| **T3** | 0.756 | 0.588 | **0.009** | **0.002** | **0.013** | **0.012** | 0.112 | - |

| **(D) Non-UV treatment pairwise comparisons for *L. calcarifer* during *S. iniae* infection at winter seasons 2020** | | | | | | | | |
| --- | --- | --- | --- | --- | --- | --- | --- | --- |
| **Time** | **T0** | **T1** | **T1-2** | **T1-3** | **T1-5** | **T2** | **T2-2** | **T3** |
| **T1** | 0.060 | - | - | - | - | - | - | - |
| **T1-2** | **0.015** | **0.046** | - | - |  |  |  | - |
| **T1-3** | 0.761 | 1.000 | 1.000 | - | - | - | - | - |
| **T1-5** | 1.000 | 1.000 | 1.000 | 1.000 | - | - | - | - |
| **T2** | 1.000 | 1.000 | 1.000 | 1.000 | 1.000 | - | - | - |
| **T2-2** | 1.000 | 1.000 | 1.000 | 1.000 | 1.000 | 1.000 | - | - |
| **T3** | 1.000 | 1.000 | 1.000 | 1.000 | 1.000 | 1.000 | 1.000 | - |

| **(E) UV treatment pairwise comparisons for *L. calcarifer* during *S. iniae* infection at winter seasons 2020** | | | | | | | | |
| --- | --- | --- | --- | --- | --- | --- | --- | --- |
| **Time** | **T0** | **T1** | **T1-2** | **T1-3** | **T1-5** | **T2** | **T2-2** | **T3** |
| **T1** | 1.000 | - | - | - | - | - | - | - |
| **T1-2** | 0.375 | 1.000 | - | - |  |  |  | - |
| **T1-3** | **0.016** | 1.000 | 1.000 | - | - | - | - | - |
| **T1-5** | 0.332 | 1.000 | 0.500 | 0.225 | - | - | - | - |
| **T2** | 0.321 | 1.000 | 1.000 | 1.000 | 1.000 | - | - | - |
| **T2-2** | 1.000 | 1.000 | 0.500 | 0.680 | 1.000 | 1.000 | - | - |
| **T3** | 1.000 | 1.000 | 1.000 | 1.000 | 1.000 | 1.000 | 1.000 | - |

**Supplementary Table S9** adonis-pairwise significance test between survived and died fish skin microbiota at different body site and sampling time points for *S. aurata* during *V. harveyi* infection at winter and summer seasons in non-UV and UV treatment (A), for *S. aurata* and *L. clarifier* during *S. iniae* infection at non-UV (B).

| **(A) Pairwise comparisons between those survived and died fish for *S. aurata* during *V. harveyi* infection at winter and summer seasons** | | | | | | | |
| --- | --- | --- | --- | --- | --- | --- | --- |
| **Season** | **Summer 2015** | | | **Winter 2016** | | | |
|  | **Non-UV** | | | **Non-UV** | | | **UV** |
|  | **T0** | **T1** | **T2** | **T0** | **T1** | **T2** |  |
| **Survived vs died** | A = 0.324 G = 0.533  L = 0.853 | A = 0.095  G = 0.764  L = 0.574 | Only survived | A = 0.750 G = 0.800  L = 0.524 | A = 0.107  G = 0.071  L = 0.145 | All survived | All died |

| **(B) Non-UV treatment pairwise comparisons for *S. aurata* and *L. calcarifer* during *S. iniae* infection at winter seasons 2020** | | | | |
| --- | --- | --- | --- | --- |
|  | ***S. aurata*** | | ***S. iniae*** | |
| **Tank** | **Non-UV** | **UV** | **Non-UV** | **UV** |
| **Survived vs died** | T0 = NA  T1 = 0.196  T1-2 = 0.456  T1-3 = NA  T1-5 = NA  T2 = 0.333  T2-2 and T3 only survived available | All fish survived | T0 = NA  T1 = 0.700  T1-2 = NA  T1-3 = 0.400  T1-5 = 0.943  T2, T2-2 and T3 only survived available | T0 = 0.571  T1 = 0.167  T1-2 = 0.125  T1-3 = 1.000  T1-5 = NA  T2, T2-2 and T3 only survived available |
